# The Development of DNA Markers to Resolve Uncertainties of Seabird Bycatch Identification From Longline Fisheries in Australian Waters

**DOI:** 10.1002/ece3.70568

**Published:** 2024-11-19

**Authors:** Andrea M. Polanowski, Anna J. MacDonald, Mike C. Double, Jonathon H. S. Barrington, Theresa M. Burg, Barbara Wienecke, Julie C. McInnes

**Affiliations:** ^1^ Australian Antarctic Division, Department of Climate Change, Energy The Environment and Water Kingston Kingston Tasmania Australia; ^2^ University of Lethbridge Lethbridge Alberta Canada; ^3^ Institute for Marine and Antarctic Studies University of Tasmania Hobart Tasmania Australia

**Keywords:** albatrosses and petrels, genetic markers, longline fisheries, seabird bycatch

## Abstract

Incidental mortality in fisheries is a major driver of population declines for albatrosses and petrels globally. However, accurate identification of species can be difficult due to the poor condition of bycaught birds and/or visual similarities between closely related species. We assessed three genetic markers for their ability to distinguish the 36 albatross and petrel species listed in Annex 1 to the Agreement on the Conservation of Albatrosses and Petrels (ACAP) and in Australia's Threat Abatement Plan (TAP) for the bycatch of seabirds during oceanic longline fishing operations. We generated 275 new sequences, from 29 species, to improve the coverage of reference databases for these listed species. The combined use of the selected Cytochrome b and Control Region markers enabled the identification of 31 of 36 listed seabirds to species level and four to sister species. One petrel species could not be evaluated as no reference sequences were available. We tested these markers on 59 feathers from bycaught seabirds and compared these to onboard visual identification. We successfully assigned all procellariiforms to species (*n* = 58), whereas only two seabirds were correctly identified to species visually onboard, highlighting the difficulty of visual species assignment and the need for alternative methods. We assessed the utility of our two chosen markers for the assignment of all procellariiform species, with 74% of species with reference sequences identified to species or sister species level. However, a precautionary approach is needed for application beyond our listed species due to unvalidated reference sequences. The approach described here provides a streamlined framework for the molecular identification of seabird bycatch. This approach is recommended for use in fisheries within and outside Australian waters to improve the resolution of bycatch reporting and to corroborate logbook entries, observer reports and audits of images captured by electronic monitoring systems as well as help inform conservation efforts.

## Graphical abstract

The development of genetic methods and resources to identify seabirds threatened by fisheries bycatch. This has the potential to significantly augment existing bycatch monitoring methods and improve confidence in the understanding of species‐level impacts by specific fisheries.

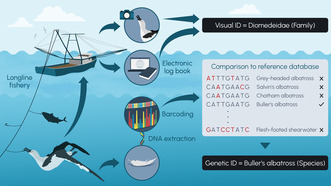

## 1 Introduction

**FIGURE 1:**
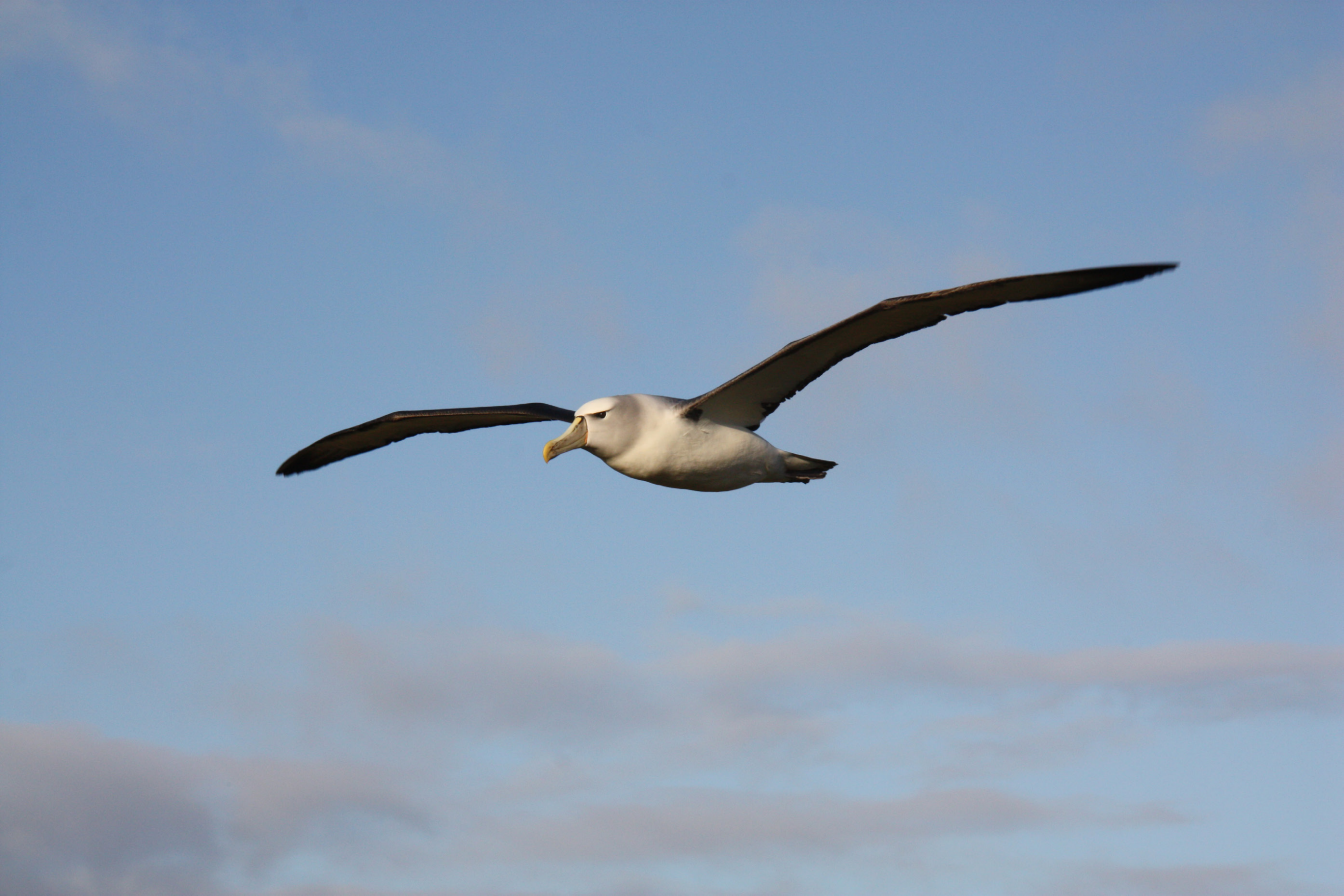
A shy albatross in flight ( *Thalassarche cauta* ). Photo: Julie McInnes.

Incidental seabird bycatch in fisheries is a significant issue globally and one of the biggest threats facing seabird populations, particularly for albatrosses, shearwaters and larger petrels (Phillips et al. 2016; Dias et al. 2019; Rodríguez et al. 2019). Fifteen of the 22 albatross species (family Diomedeidae, see Figure 1 for an example) are threatened with extinction, the highest proportion for any bird family (IUCN 2023). Effective development and evaluation of seabird bycatch mitigation requires precise information about which species comprise the bycatch. The Food and Agricultural Organisation (FAO) of the United Nations' best practice guidelines for reducing seabird bycatch in fisheries include the need to conduct independent and effective monitoring programmes (FAO 2009). Species identification is typically carried out by fisheries observers on board fishing vessels, using detailed species field guides (ACAP and NRIFSF 2015), retention of carcasses for necropsy, photography of dead animals for identification by experts and electronic monitoring using image capture and subsequent auditing (FAO 2009). However, discrepancies still exist due to difficult conditions at sea, interspecific phenotypic similarities (particularly of juvenile birds), poor specimen condition, and the prohibitive costs associated with the transport and storage of samples where more detailed analyses are required.

DNA barcoding enables species identification using suitably validated DNA sequences (Staats et al. 2016). Feather samples from seabird bycatch provide easily collectible and transportable samples for genetic analyses to facilitate species identification. DNA from feathers can be degraded, which prevents the amplification of longer PCR amplicons (Presti et al. 2013). The amplification of shorter DNA fragments (< 400 base pairs (bp)) increases the probability of success from degraded samples, such as feathers (Staats et al. 2016). However, there has been limited assessment of the applicability of shorter DNA fragments for high‐level resolution of albatross and petrel species, and it is unclear to what extent closely related species can be differentiated using shorter sequences from various gene regions.

Accurate genetic species identification relies on the presence of high‐quality reference DNA sequences in public databases and suitable DNA markers for species identification. Generally, these databases provide reference sequences from specimens with accurately assigned taxonomy (ideally from known provenance animals). To enable identifications, DNA sequences from specimens of unknown origins are usually compared to reference sequences from known specimens deposited in such databases, via alignment searching (BLAST) or distance‐based tree construction. A suitable marker for identification at the species level should be sufficiently variable between species (interspecific variation) and ideally display either low or no variation within species (intraspecific variation) (Staats et al. 2016). Markers should be well‐characterised for a large number of species to enable reliable comparisons. The discriminating power of these methods is directly related to the prior choice of markers and the reference database quality and completeness. Importantly, a reference database should include multiple sequences from each species within the taxonomic group of interest and multiple individuals per species at multiple populations/breeding sites (MacDonald and Sarre 2017) to accurately estimate inter‐ and intraspecific variation for each chosen marker.

In the past 20 years, a variety of molecular markers and methods, each with their own strengths and weaknesses, have been used to determine the origin and/or identification of seabird bycatch specimens. Studies have focused on a limited number of seabird species, often with a single mitochondrial marker, and used high‐quality DNA from tissue (e.g. Walsh and Edwards 2005; Techow et al. 2016; Abbott et al. 2006). The mitochondrial control region (CR) has been used to distinguish between bycatch from several albatross species (Abbott et al. 2006; Burg 2007; Jiménez et al. 2009, 2015; Wold et al. 2018). Provenance of bycaught specimens has been investigated with microsatellites in albatrosses (Abbott et al. 2006; Burg 2007, 2023) and northern fulmars using restriction‐site associated DNA sequencing (RADseq; Baetscher et al. 2022) However, as with most of the studies mentioned above, reference data from known provenance populations are required to provide baseline data for these markers. Furthermore, no studies have successfully tested markers across multiple families of seabirds to enable the detection of albatrosses, shearwaters and petrels. Improved reference databases are essential to expanding from single‐species studies to cross‐family analysis.

Since 1998, Australia has implemented successive threat abatement plans (TAP) for the incidental catch (or bycatch) of seabirds during oceanic longline fishing operations (TAP‐Seabirds, Commonwealth of Australia 2018). The threat abatement plan applies to all Australian Commonwealth‐managed oceanic longline fisheries within Australia's jurisdiction. This requires data to be collected on bycatch and prioritises accurate species determination. However, of the 282 dead or injured seabirds reported as bycatch in all Australian Commonwealth oceanic longline fisheries between 2019 and 2022, species‐level identification was assigned for only 30% (*n* = 85; Threatened and Endangered Species Reports (TEP), 2019–2022; AFMA 2023). The remaining samples were grouped into broad categories, such as ‘albatross’ or ‘bird’, which does not allow for a full assessment or quantification of the impact of seabird interactions with fishing operations at species or population levels or meet the needs of the TAP which specifies the need to identify albatross and other seabird species affected by the key threatening processes.

Efforts to improve species identification in three oceanic longline fisheries: Eastern Tuna and Billfish Fishery (ETBF), Western Tuna and Billfish Fishery (WTBF), and the Gillnet, Hook and Trap Sector (GHAT) of the Southern and Eastern Scalefish and Shark Fishery (SESSF) have included the implementation of the Seabird Feather Kit Collection Program (SFKCP). In the event of a seabird interaction that results in mortality, longline fishers hold the bird in front of electronic cameras, record the interaction in an electronic logbook (e‐log) and collect feather samples for genetic analysis based on the guide developed by the Agreement of the Conservation of Albatrosses and Petrels (ACAP) and the National Research Institute of Far Seas Fisheries (ACAP and NRIFSF 2015).

This work to develop genetic markers for species identification was motivated by recognition of the difficulties associated with identifying many seabird species in the field, especially phenotypically similar species and/or degraded specimens. This work is not intended to be critical of the identification skills of fishers but rather aims to improve confidence in our knowledge of the species caught by providing an additional line of evidence. The aims of this study were to provide a genetic method, optimised for application to degraded samples, that allows for the identification of albatross, petrel and shearwater species caught as fishery bycatch in Australian waters, and tested the utility of the methods more broadly. To achieve these aims, we (1) identify DNA markers for species identification of the 36 albatross, shearwater and petrel species listed in Annex 1 to ACAP and in Australia's TAP‐Seabirds (hereafter referred to as listed species) suitable for degraded samples, (2) assess reference database coverage for those markers and where possible expand this to include all listed species, (3) evaluate the utility of the markers included in this study for species identification, in a broader context, for all other procellariiform species and (4) demonstrate the implementation of those markers by determining the species composition of fisheries bycatch carcasses recovered from Australian oceanic longline fishing vessels from 2019 to 2022. Overall, this project moves towards developing a standardised approach to identifying the listed bycatch species using custom DNA reference databases and takes the first steps towards a molecular framework for detecting procellariiform bycatch globally.

## 2 Materials and Methods

### 2.1 Species Included in This Study

**FIGURE 2:**
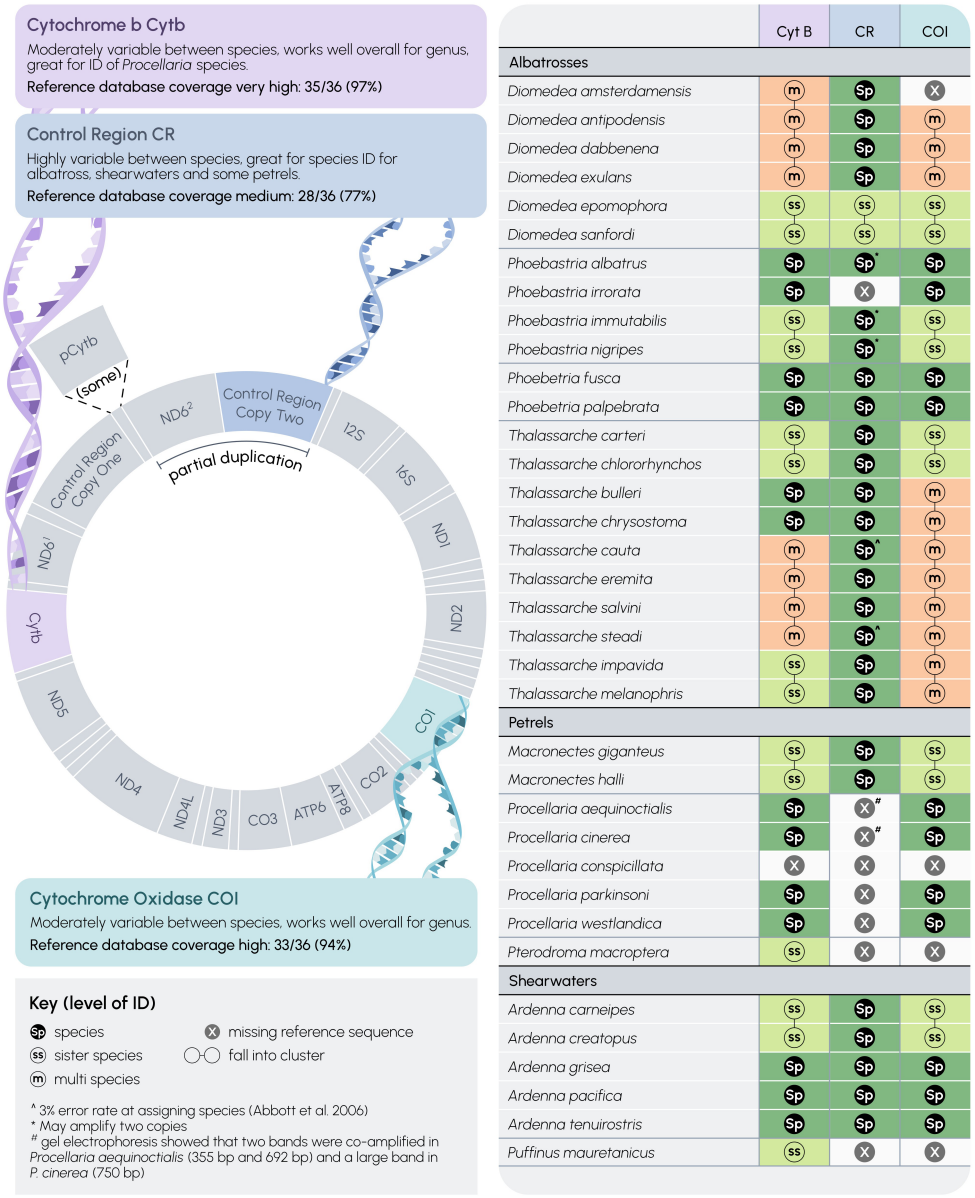
Species resolution for the three tested primer sets for the 36 listed procellariiform species. Dark green shading indicates unknown sequences can be identified to species, light green indicates unknown sequences can be identified to sister species, and orange indicates unknown sequences can be identified to multiple species. Key: S in the black circle indicates unknown sequences can be identified to species, SS in the grey circle indicates unknown sequences can be identified to sister species, m in the grey circle indicates unknown sequences can be identified to multiple species, x in the grey circle indicates a missing reference sequence, two circles joined by a line indicates the sample falls into a cluster of multiple species. (Illustrated by Stacey McCormack [Visual Knowledge Pty Ltd]).

To address specific Australian management aims to identify seabirds caught in Australian waters, we focused on 36 species within the order Procellariiformes (hereafter referred to as the listed species, Figure 2) that include the 22 albatross and nine petrel species listed in ACAP Annex 1 to the Agreement (www.acap.aq) and an additional five species of petrels and shearwaters (*Ardenna* and *Pterodroma* spp.) listed in Annex A to the TAP‐Seabirds (Commonwealth of Australia 2018). The included species were those assigned under the ACAP Taxonomy Working Group and IOC World Bird List (Gill, Donsker, and Rasmussen 2023), respectively. Currently, ACAP does not consider Antipodean and Gibson's albatross ( *Diomedea antipodensis* and *D. gibsoni* ) as separate species, and therefore these taxa were subsumed under the single species Antipodean albatross ( *D. antipodensis* ) in this study. For the listed species, we determined the availability of existing mitochondrial reference DNA sequences, identified three genetic markers suitable for sister species and species identification, generated new reference sequences from specimens of known provenance and generated a custom reference sequence database for each marker. We outline a framework for the application of these genetic markers to identify unknown specimens, illustrated with a case study from an Australian fishery.

The 36 listed species represent only a proportion of Procellariiformes, a diverse order of seabirds that includes albatrosses, petrels, shearwaters, storm petrels and diving petrels. To evaluate the potential for application of our framework to species identification in a broader international context, and to determine the risks of misidentification between our listed species and other procellariiform species, we also constructed custom reference sequence databases and evaluated the three markers using all available sequences from the order Procellariiformes.

### 2.2 Marker Selection, Primer Design and Laboratory Evaluation of Primers

Mitochondrial DNA (mtDNA) has been extensively used to study the molecular diversity of procellariiforms (Burg and Croxall 2004; Jesus et al. 2009; Welch, Olson, and Fleischer 2014). Such application of mtDNA assumes that each marker is single copy, but in fact partial duplication of the mitochondrial genome is widespread within procellariiforms (see Torres et al. 2019). Therefore, caution needs to be exercised when designing primers for mtDNA to avoid co‐amplification of paralogues (Torres, Bretagnolle, and Pante 2022). In this study, we evaluated six primer pairs (Table A1) from three mitochondrial regions: two for Cytochrome Oxidase I (COI), three for Cytochrome b (Cytb) and one for the Control Region (CR). These included primers designed or modified for this study by aligning and manually inspecting mtDNA sequences retrieved from the NCBI database for the 36 listed species to identify conserved regions suitable for primer design (development of reference DNA sequence databases to inform primer design is outlined further in Section 2.3).

An approximately 650 bp region of the mitochondrial Cytochrome *c* Oxidase I gene (COI) is used as the standard DNA barcoding marker for most animals (The International Barcode of Life Consortium 2023). However, the full length of the COI marker may be difficult to recover from degraded DNA samples. Two COI primer pairs were chosen for evaluation, using the same universal reverse primer jgHCO2198 (Geller et al. 2013), which includes inosine nucleotides (a DNA base that complements all four nucleotides) to increase amplification success across a broad spectrum of metazoan phyla. The first primer set, pairing jgHCO2198 with a modified version of AvMiF1, (used for testing the effectiveness of DNA barcodes for species identification of Neotropical birds; Kerr et al. 2009) amplifies a 466 bp fragment. The second set, pairing jgHCO2198 with BirdCOIF, (a version of Sauron‐S878F, a universal COI forward primer; Rubbmark et al. (2018) modified here to improve coverage for procellariiforms), amplifies a 367 bp fragment (Table A1). Three primer pairs were chosen for evaluation for Cytochrome b (Cytb): two primer pairs that were unique to this study and an existing pair used for identifying seabirds on Macquarie Island (McInnes et al. 2021; Table 2).

The CR has an exceptionally fast evolutionary rate and is considered the most variable region of the mitochondrial genome, making it a powerful marker to resolve the phylogenetic inference of closely related species (Bronstein, Kroh, and Haring 2018). However, many procellariiform species have two (non‐identical) copies of the CR (Abbott et al. 2005; Eda et al. 2010; Burg et al. 2014; Lawrence, Lyver, and Gleeson 2014; Torres et al. 2019) that can be co‐amplified by PCR. The duplicated CRs have been sequenced for five *Thalassarche* (Abbott and Double 2003b; Abbott et al. 2005) and two *Diomedea* albatrosses (Rains, Weimerskirch, and Burg 2011) resulting in the development of two PCR primer pairs, SPECF1 and SPECF2 (Abbott and Double 2003b), that specifically amplify the first domain of copy 1 and copy 2, respectively. The highly variable nature of the CR and the complex inheritance of the duplicated regions in procellariiforms (Torres et al. 2019) prevented the development of a set of ‘universal procellariiforms’ CR primers (see Table A2 for the full alignment of the F1 and F2 copy in the forward primer in available sequences). In this study, we modified the SpecF2/GluR7 primers to also amplify the CR copy 2 markers for flesh‐footed (*Ardenna carneipes*) and pink‐footed (*A. creatopus*) shearwaters. The SpecF2 primer was modified at the 3′ end, with the removal of two As to increase binding in *Ardenna* species, and modified at the 5′ end with the addition of GCA, which was conserved among all species. The remaining part of the primers was not modified, and degenerate bases were not included in case they introduced bias over which copy was amplified (CRBird_F and CRBird_R primers; Table A1).

To evaluate the applicability of the primers, we tested the ability of each candidate primer set (Table A1) to amplify DNA from tissue from 10 listed species (*Ardenna carneipes*, *Diomedea antipodensis* , *D. exulans* , *Phoebetria palpebrata* , *Thalassarche bulleri* , *T. carteri* , *T. cauta* , *T. impavida* , *T. salvini* and *T. steadi*). Additionally, to test the applicability of the primers to specimens at varying grades of preservation, we tested PCR amplification success from DNA extracted from > 12 feathers. Based on these evaluations, we selected one primer pair for each marker (Table 1; henceforth each marker is referred to as COI_AP, Cytb_AP and CRBird_AP).

**TABLE 1:** The three primer pairs selected for use in this study, following initial evaluation, including PCR product length and amplification temperature.

Locus	Primer Name	Primer Sequence	PCR Temp	Length (bp)	Reference
COI	BirdCOIF	GGNACMGGRTGRACHGTNTAYCCNCC	45°C	367	Geller et al. (2013), Rubbmark et al. (2018)
	jgHCO2198R	TAIACYTCIGGRTGICCRAARAAYCA			
CR (Copy 2	CRBird_F	CAGCCTATGTGTTGATGTGCA	50°C	379	This study Modified from Abbott and Double (2003b)
Domain1)	CRBird_R	CGGGTTGCTGATTTCTCGTG			
Cytb	Cytb2‐F	TAYATYGGCCARACCYTYGTAG	53°C	305	McInnes et al. (2021)
	Cytb2‐R	GTTYTCTGGRTCDCCKARYA			

*Note:* Underlined bases are modifications to the original primer Sauron‐S878F.

For two closely related species with minimal genetic differences (shy and white‐capped albatross), we also explored two sex‐linked markers, the mitochondrial 16S gene and 23 nuclear markers for fixed genetic differences between the species (Table A3).

### 2.3 Development of Custom Reference DNA Sequence Databases for Listed Species and for All Procellariiforms

Correct taxonomic assignment of the listed species depends on the existence and the quality of genetic databases (Conde‐Sousa, Pinto, and Amorim 2019). Reference DNA sequences should ideally be sourced from samples of known provenance (e.g. samples collected from breeding sites) that have reliable taxonomic identification. We assessed the availability of procellariiform mitochondrial DNA reference sequences from the NCBI GenBank database.

Initially, to inform primer design and evaluation of the six genetic markers described above, we retrieved all available COI, CR and Cytb sequences for the 36 listed species (GenBank accessed in March 2023). For each gene region, sequences were aligned in Sequencher (version. 4.10.1) and manually inspected for conserved regions. Following marker selection, we identified gaps in reference sequence coverage for the 36 listed procellariiform species. To address these data gaps, we sourced 99 reference samples (Table S1) from DNA, tissue, blood or feathers. DNA was extracted from 18 museum samples and five feather samples using the Qiagen DNeasy Blood & Tissue kit (Qiagen), with modifications based on Joseph et al. (2016). DNA from a total of 84 reference DNA samples was sequenced using the three selected primer pairs (Table 1). Reference DNA sequences for an additional 15 samples were obtained through collaboration with B. N. Sacks, from the University of California, and his colleagues E. Pulido and S. Vanderzwan. Sequences were trimmed, edited and aligned using GeneiousPrime 2022.0.1 (https://www.geneious.com) and queried (blastn) against the National Center for Biotechnology Information (NCBI) nucleotide database to confirm the identification of each sequenced PCR product.

To enable evaluation of the three selected markers across all procellariiform species, we subsequently developed a custom reference DNA sequence database for each marker (COI_AP, Cytb_AP and CRBird_AP), using all available sequences from all 149 procellariiform species. These databases included all relevant procellariiform sequences from GenBank (families Diomedeidae, Hydrobatidae, Oceanitidae and Procellariidae; accessed July 2023), sequences extracted from the mitochondrial genomes of four North Pacific albatross species (genus *Phoebastria*) and for wandering albatross ( *D. exulans* ) as assembled by Huynh et al. (2023), and the new reference sequences generated in this study (described above). Two CR copy two sequences, previously unpublished by Rains, Weimerskirch, and Burg (2011), were also included (Table S1; *Diomedea exulans* ; PP712121 and PP712122). Further details on the development of the custom procellariiform reference databases are provided in Appendix 1.

### 2.4 In Silico Evaluation of Markers for Identification of Listed Species and All Procellariiform Species

We used a genetic distance‐based method to evaluate the utility of the three selected genetic markers for species‐level identification of the 36 listed species. We also evaluated the three markers for potential broader application to identify all procellariiform species. We used the R package SPIDER (Brown et al. 2012) to evaluate the three markers; using the custom databases, we had developed for all procellariiform species (described above) as the three input data files. Species with only one unique haplotype were included in these analyses, but intraspecific genetic distances cannot be evaluated for these species, which limits some interpretation of the results. For each marker, pairwise genetic distance was calculated for each pair of sequences using the ‘raw’ or uncorrected model (Collins et al. 2012; Srivathsan and Meier 2012). We then analysed each database using the *threshID* function to identify instances where a risk of species misidentification or ambiguity was likely, and to identify genetic distance thresholds that might be used to guide the assignment of DNA sequences of unknown provenance to a species or genus (Appendix 1). From this, we determined the proportion of species that could be assigned to species or sister species for each marker, both for the Procellariiforme order overall as well as within each of the four procellariiform families.

### 2.5 Case Study: Genetic Identification of Listed Species From Bycatch Feather Samples

Feather samples were collected from 59 seabirds caught incidentally from 2019 to 2022 (56 in the ETBF and 3 in the GHAT sector of the SESSF). Multiple feathers were plucked from each deceased bird following established protocols and stored at −20°C until DNA could be extracted. AFMA e‐log records were available for the 59 feathers, which include seabird identifications based on visual observation by the fishery operators.

DNA from the feather samples was amplified and sequenced using either all three markers (COI_AP, CRBird_AP and Cytb_AP, *n* = 20 feathers) or just the two markers recommended based on results of the initial trials (Cytb_AP and CRBird_AP, *n* = 39 feathers). For each of the bycatch specimens (*n* = 59), sex was also determined by analysis of feather DNA using a real‐time melt curve analysis (Faux, McInnes, and Jarman 2014). Further details of the bycatch feather DNA extractions, PCR amplification, sequencing and sexing methods are provided in Appendix 1.

## 3 Results

### 3.1 Marker Selection, Primer Design and Laboratory Evaluation of Primers

We evaluated six primer pairs from three mitochondrial regions. DNA from 10 tissue and > 12 feather samples, representing 10 listed species, was used in an initial experiment to identify the optimal primer sets to use for species identification. All six primer pairs were tested in vitro, and three primer pairs were selected for use in this study (Table 1; see Table A1 for full details, including reasons for primer exclusion).

Considering the three selected markers, COI_AP amplified all 10 tissue samples successfully and 16/20 feather samples. Cytb_AP worked in all 10 tissue and 20 feather samples. However, an approximately 160 bp section of the duplicated Cytb_AP region was co‐amplified using the Cytb_AP marker in the three *Phoebastria* species, although no mixed bases were present in any of the sequences (identified by BLAST search). Finally, CR_Bird_AP amplified all 10 tissue samples and 18 out of 20 feathers, but we encountered some evidence of potential duplication of this region during subsequent sequencing efforts. We attempted to amplify the CRBird_AP marker from white‐chinned ( *Procellaria aequinoctialis* ) and grey petrels ( *P. cinerea* ), to generate reference DNA sequences for these species, but gel electrophoresis showed either two bands were co‐amplified (355 and 750 bp; *Procellaria aequinoctialis* ) or one large band (750 bp, *P. cinerea* ). Further, in the North Pacific albatross species (*Phoebastria*), the CRBird_AP marker seemed to preferentially amplify control region copy 1 in our laboratory analyses: the primers only amplified copy 1 in short‐tailed albatross (*P. albatrus, n* = 3); in Laysan albatross ( *P. immutabilis* ,*n* = 5), the primers amplified CR copy 1 alone from four samples but amplified both CR copies from the fifth sample; in black‐footed albatross (*P. nigripes, n* = 7), the primers amplified CR copy 1 from five samples and CR copy 2 from two samples. A possible explanation for this is that there are more mismatches to the CRBird reverse primer in the F2 copy than in the F1 copy in this genus (see Table A2 for the full alignment of the F1 and F2 copies).

### 3.2 Development of Custom Reference DNA Sequence Databases for Listed Species and for All Procellariiforms

Reference DNA sequence databases for three mitochondrial gene regions were constructed using all relevant sequences available from GenBank in March 2023, to evaluate genetic markers for our listed species. Several gaps in coverage of these reference databases were identified. A total of 996 procellariiform COI sequences were available overall, but these covered only 23 of our 36 listed species (64%), including only 11 of the 22 albatross species. Universal primers for Cytb became available long before COI and consequently, GenBank contains several thousand Cytb sequences from a large range of species (Staats et al. 2016). In March 2023, these included 1921 procellariiform Cytb sequences, and the entire Cytb gene (~1140 bp) had been sequenced for 35 of the 36 listed seabird species. However, for 18 of the listed species (50%), only a single Cytb sequence was available, presenting an incomplete picture of intraspecific genetic diversity. Finally, reference sequences for the CR marker were only available for 15 of the 36 listed species.

To address these gaps in the reference database coverage for our listed species, we sourced 99 reference samples, representing 29 of the 36 listed species, and generated 275 new sequences; 96 for COI_AP, 95 for Cytb_AP and 84 for CRBird_AP (Table S1, GenBank accession numbers: COI_AP PP412076—PP412170, Cytb_AP PP447552—PP447646, CRBird_AP PP447647—PP447727).

Following the selection of three genetic markers for further evaluation (COI_AP, Cytb_AP, and CRBird_AP) and the generation of new reference DNA sequences as part of this study, in July 2023 we re‐assessed the availability of reference sequences for those three markers for all procellariiform species (*n* = 149 species across 26 genera). The custom reference DNA sequence database developed for each marker included sequences from GenBank and sequences generated in this study (Table S2, SuppInfo_COI_AP, SuppInfo_Cytb_AP, SuppInfo_CRBird_AP). Overall, Cytb_AP had the greatest species coverage, with reference sequences available for 89% of all procellariiform species (*n* = 133), compared to 62% (*n* = 93) for COI_AP and 25% (*n* = 38) for CRBird_AP (Table 2). Cytb_AP also had the highest average number of sequences per species (Table A4). Further, these custom databases now provide reference sequences for the majority of the listed species (a least one sequence available for 97% of listed species for Cytb and CR respectively, and 92% for COI), providing a strong foundation for the assignment of ACAP species in bycatch (Table 2).

**TABLE 2:** Summary of the number of species within each procellariiform family for which reference sequences are available, and the utility of these for identification of unknown sequences to species or sister‐species level, for each of the three markers used in this study.

	Total number of species	COI_AP			Cytb_AP			CRBIRD_AP			Cytb_AP and/or CRBIRD_AP		
		Species with ref. sequence	Species ID	Sister‐species ID	Species with ref. sequence	Species ID	Sister‐species ID	Species with ref. sequence	Species ID	Sister‐species ID	Species with ref. sequence	Species ID	Sister‐species ID
Listed species	36	33 (92%)	11 (33%)	10 (30%)	35 (97%)	13 (37%)	12 (34%)	28 (78%)	26 (72%)	2 (7%)	35 (97%)	31 (89%)	4 (11%)
Diomedeidae	22	21	4	6	22	6	8	21	19	2	22	20	2
Procellariidae	99	58	38	12	87	44	15	14	11	0	87	48	11
Hydrobatidae	18	8	8	0	17	9	2	3	0	0	17	9	2
Oceanitidae	10	6	6	0	7	7	0	0	0	0	7	7	0
All procellariiform species	149	93	56	18	133	67	25	38	30	2	133	84	15

*Note:* For some genera, at least one species has no reference DNA sequence available: Identifications to species or sister‐species level should be revised if reference sequences representing new species are added to the database in the future.

### 3.3 In Silico Evaluation of Markers for Identification of Listed Species and All Procellariiform Species

We evaluated the utility of the three selected genetic markers for species‐level identification of the 36 listed species (Figure 2, Table S3). The COI_AP marker was the least successful for species identification. COI_AP reference sequences were available for 33 of 36 listed species, but only 11 (33%) of these could be identified as species and an additional 10 (30%) as sister species. Cytb_AP reference sequences were available for 35 of 36 listed species. The Cytb_AP marker provided species‐level resolution for 13 (37%) of these and resolution to sister species for another 14 (40%). CRBird_AP reference sequences were available for 28 of the 36 listed species. The CRBird_AP marker provided species‐level resolution for 26 (93%) of these and an additional two (7%) for sister species (Table 2).

The utility of the markers for determining species identification varied among families. The COI_AP marker provided insufficient resolution between closely related albatross species, with only four of the 22 albatrosses identified as species (18%). The Cytb_AP marker identified six of the 22 albatrosses to species (27%) and eight to sister species. The Cytb_AP marker provided resolution for petrels and shearwaters, with seven of the 14 identified to species (50%) and six to sister species (43%). The CRBird_AP marker was more accurate for southern hemisphere albatrosses, with 16 of the 18 albatross species identified to species level (89%), and only northern and southern royal albatross ( *Diomedea epomophora* and *D. sanfordi* ) unresolved (Figure 2, Table S3).

Despite some uncertainty around the preferential amplification of CR copy 1 in the genus *Phoebastria*, CRBird_AP sequences obtained from three *Phoebastria* species here and in previous studies were still useful for species identification. We recommend using this marker with caution for *Phoebastria*: combined use with the Cytb_AP marker will increase confidence in interpretation. Seven of the 14 listed petrel and shearwaters species (50%) were identified to species level using the CRBird_AP marker. The CRBird_AP marker could not be evaluated for six listed species: the waved albatross ( *Phoebastria irrorata* ), Balearic shearwater ( *Puffinus mauretanicus* ), great‐winged petrel ( *Pterodroma macroptera* ) and three *Procellaria* species, as no reference sequences were available for these taxa (Figure 2, Table S3). Shy and white‐capped albatrosses are closely related species that are difficult to distinguish morphologically and genetically (Abbott and Double 2003b). Previous work, with SpecF2 and GluR7 primers, identified a single nucleotide polymorphism (SNP) in CR copy 2, domain 1, that distinguishes these two species in almost all cases (Abbott and Double 2003b). The same SNP site is conserved when amplified with the CRBird_AP primers and provides 97% accuracy in assigning species (Abbott et al. 2006).

**FIGURE 3:**
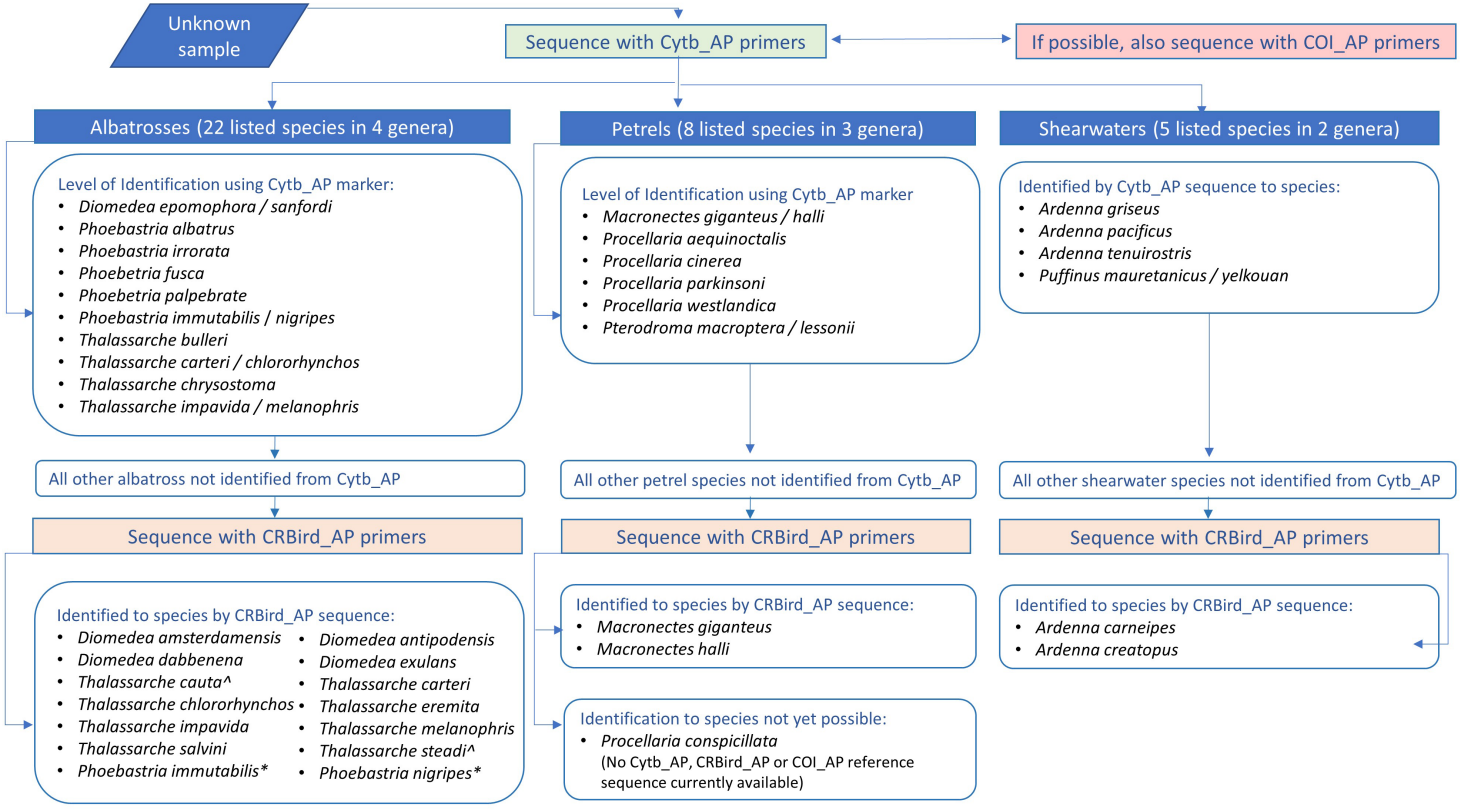
Decision tree for highest species level identification using the Cytb_AP and CRBird_AP markers. *May amplify two copies; ^^^ Species identification was based on a single nucleotide polymorphism (SNP) in the mitochondrial control region (Abbott and Double 2003b). This method has a ~3% error in assigning species (Abbott et al. 2006).

No single marker was able to identify all of the listed birds to species; however, species resolution was significantly improved when both the Cytb_AP and CRBird_AP markers were used in combination. We recommend the use of the Cytb_AP marker initially and then the CRBird_AP marker if needed for species‐level identification (Figure 3). In combination, these two genetic markers identified 31 (86%) of our listed species to species level, and an additional four (11%) to sister species (Table 2). The four listed species that could not be resolved beyond the sister species level were northern and southern royal albatross, great‐winged petrel (match to white‐headed petrel [ *P. lessonii* ], not one of our listed species), and Balearic shearwater (match to Yelkouan petrel [ *P. yelkouan* ], not one of our listed species). We were unable to evaluate the reliability of the identification of one of our listed species, the spectacled petrel ( *Procellaria conspicillata* ), as no reference DNA sequences are currently available for any of our three markers, and we were unable to source suitable samples to generate new sequences.

We used genetic‐distance‐based threshID analyses to evaluate each marker using all available procellariiform reference sequences (Appendix 2). At the genus level, using a genetic distance threshold of 4% (COI_AP and Cytb_AP, equivalent to sequences sharing ≥ 96% identity) or 7% (CRBird_AP, equivalent to sequences sharing ≥ 93% identity), almost all sequences were assigned to the correct genus (Table A5). At the species level, across all three markers, a threshold of 1.5% had the lowest risk of errors (equivalent to sequences sharing ≥ 98.5% identity). While many sequences were correctly assigned to species using a 1.5% threshold, ambiguous, incorrect or ‘no identification’ results were observed for all three markers (Table A5). Using a 1.5% threshold, 60% (*n* = 56) of species with a reference sequence could be assigned to species for CO1_AP, 74% (*n* = 28) for CRBird_AP and 49% (*n* = 66) Cytb_AP. When considering all procellariiforms, as for the listed species, the utility of the markers for determining species identification varied among families. We recommend using a combination of CRBird_AP and Cytb_AP for procellariiform species identification more broadly: using these two markers in combination, we could assign 63% of species with a reference sequence (*n* = 84) to species level, and another 11% (*n* = 15) to sister species (Table 2). However, for some genera, a combination of Cytb_AP and COI_AP may be more useful, as the availability of CRBird_AP reference DNA sequences is currently limited for many procellariiforms (Table A6). For applications beyond our case study, the most appropriate genetic markers should be selected based on consideration of the available reference data. Overall, these results demonstrate that the markers have different utility depending on the families and highlight the importance of improving the coverage of intraspecific genetic variation within the reference databases to evaluate barcoding utility more comprehensively. The usefulness of these markers may also differ between geographic locations. For example, identification to species level may be improved by excluding species that do not occur in the study range.

### 3.4 Case Study: Genetic Identification of Listed Species From Bycatch Feather Samples

Of the 59 bycatch feather samples analysed with the Cytb_AP and CRBird_AP marker, 58 were from albatrosses or petrels and could be genetically identified as species, with nine species identified (Table 3). The most commonly detected species from the feather samples were flesh‐footed shearwater (*Ardenna carneipes*, *n* = 27, 46%) with 52% from females and 48% from males, and Antipodean albatross ( *Diomedea antipodensis* , *n* = 18, 31%), 71% were from males and 29% from females. Antipodean albatross samples were almost entirely from 2022. In addition to the 18 assigned to Antipodean albatross, another 10 feather samples were assigned to the family Diomedeidae; six from white‐capped albatross (*Thalassarche steadi*), two from Buller's albatross ( *T. bulleri* ), one from Campbell albatross ( *T. impavida* ), and one from wandering albatross ( *D. exulans* ). Three feather samples originated from white‐chinned petrel ( *Procellaria aequinoctialis* ). One feather was from a non‐listed species (tern) and was identified to *Sterna* sp. (see Table A7 for a summary of bycatch feather samples and SuppInfo_Bycatch_Feather for sequences in FASTA format; COI_AP *n* = 19, Cytb_AP *n* = 59, CRBird_AP *n* = 54).

**TABLE 3:** Number of bycatch feather samples identified to species level using genetic methods.

Species	Fishery	2019	2020	2021	2022	Total	%	Sex^a^		
								F	M	U
Flesh‐footed shearwater (*Ardenna carneipes*)	ETBF	2		4	21	27	45.8	13	12	2
Antipodean albatross (*Diomedea antipodensis*)	ETBF	5			13	18	30.5	5	12	1
White‐capped albatross (*Thalassarche steadi*)	ETBF	3	2		1	6	10.2	4	2	
Buller's albatross (*Thalassarche bulleri*)	ETBF	1		1		2	3.4	1	1	
White‐chinned Petrel (*Procellaria aequinoctialis*)	SESSF				3	3	5.1	2	1	
Campbell's albatross (*Thalassarche impavida*)	ETBF			1		1	1.7	1		
Wandering albatross (*Diomedea exulans*)	ETBF				1	1	1.7		1	
Tern (*Sterna* sp.)	ETBF		1			1	1.7			1
Total number of feather samples analysed using genetic markers		11	3	6	39	59				
Number of dead/injured seabird interactions reported in the ETBF (TEP reports^b^)		78	33	42	58	211				
Number of dead/injured seabird interactions reported in the SESSF (TEP reports^b^)		NA	NA	NA	NA	20				
Percentage of dead/injured seabirds analysed using genetic markers		14%	9%	14%	67%	28%				

*Note:* Fifty‐six feather samples were collected in the Eastern Tuna and Billfish Fishery (ETBF) between 2019 and 2022 and three in the Gillnet Hook and Trap Sector (GHAT) of the Southern and Eastern Scalefish and Shark Fishery (SESSF) in 2022.

^a^ Results of genetic sexing test: F = female, M = male, U = undetermined.

^b^ TEP reports (AFMA 2023).

We compared results from visual identifications of bycaught seabirds with genetic identifications (Table 4). The e‐log records included 23 specimens visually identified to the family Diomedeidae (Albatrosses), but genetic identification was able to provide greater resolution to species level ( *Diomedea antipodensis* (*n* = 17), *D. exulans* (n = 1), *Thalassarche steadi* (*n* = 2), *T. bulleri* (*n* = 2) *and T. impavida* (*n* = 1)). Five albatross specimens were identified at the species level in the fishery e‐log books, but none of these identifications matched the genetic results at the species level. Two specimens were identified as flesh‐footed shearwater (*A. carneipes*) by both methods. The elog records identified three specimens to the family Procellariidae and 18 to the genus *Ardenna*, which was consistent with the genetic results that assigned all of the samples to species level: *A. carneipes*. Four samples that were visually identified as short‐tailed shearwaters ( *A. tenuirostris* ) were genetically identified as flesh‐footed shearwater (*n* = 1) and white‐chinned petrel ( *Procellaria aequinoctialis* ; *n* = 3). Genetic identifications were also obtained for an additional three feather samples collected during this time frame that did not have an e‐log record. The individual specimen visually identified as a tern was identified to genus with the genetic identification.

**TABLE 4:** Comparison between elog records (visual identifications) and genetic identifications of bycatch samples in the Eastern Tuna and Billfish Fishery between 2019 and 2022 (*n* = 56) and Southern and Eastern Scalefish and Shark Fishery (SESSF) Gillnet Hook and Trap Sector (GHAT) in 2022 (*n* = 3) (visual observation data provided by AFMA, extracted from e‐logbooks).

Date of Interaction(mm/yyyy)	Number of bycatch samples	Identification of bycatch specimen(s)		Resolution of identification		Agreement between elog and genetic ID		
		elog record (AFMA)	Genetics (this study)	elog	Genetics	Family level	Genus level	Species level
02/2019 09/21 & 10/2022	18	*Ardenna spp.—*undifferentiated	*Ardenna carneipes*	Genus	Species	Y	Y	n/a
04/2019 & 11/2019	2	*Diomedeidae—*undifferentiated	*Thalassarche steadi* ^a^	Family	Species	Y	n/a	n/a
05/2019	1	*Diomedea exulans*	*Thalassarche steadi* ^a^	Species	Species	Y	N	N
09/2019 & 10/2021	2	*Diomedeidae—*undifferentiated	*Thalassarche bulleri*	Family	Species	Y	n/a	n/a
10/2019 and 10/2022	17	*Diomedeidae—*undifferentiated	*Diomedea antipodensis*	Family	Species	Y	n/a	n/a
10/2019	1	*Ardenna tenuirostris*	*Ardenna carneipes*	Species	Species	Y	Y	N
03/2020	1	*Thalassarche melanophris*	*Thalassarche steadi* ^a^	Species	Species	Y	Y	N
06/2020 and 04/2022	2	*Thalassarche cauta*	*Thalassarche steadi* ^a^	Species	Species	Y	Y	N
10/2020	1	*Laridae (tern)*	*Sterna sp*.	Family	Genus	Y	n/a	n/a
06/2021	1	*Diomedeidae—*undifferentiated	*Thalassarche impavida*	Family	Species	Y	n/a	n/a
09/2021	3	*no AFMA record*	*Ardenna carneipes*	n/a	Species	n/a	n/a	n/a
10/2022	1	*Diomedeidae—*undifferentiated	*Diomedea exulans*	Family	Species	Y	n/a	n/a
10/2022	2	*Ardenna carneipes*	*Ardenna carneipes*	Species	Species	Y	Y	Y
10/2022	3	*Procellariidae*—undifferentiated	*Ardenna carneipes*	Family	Species	Y	n/a	n/a
03/2022	1	*Diomedea exulans*	*Diomedea antipodensis*	Species	Species	Y	Y	N
12/2022 (SESSF)	3	*Ardenna tenuirostris*	*Procellaria aequinoctialis*	Species	Species	Y	N	N

^a^ Discrimination between *Thalassarche cauta* and *T. steadi* based on genetic methods has 97% accuracy (Abbott et al. 2006).

## 4 Discussion

The accurate identification of seabird species is essential not only for understanding the impacts of fishery bycatch on species populations but also for improving bycatch mitigation and the sustainability of fisheries. Here we provide a genetic method, optimised for application to degraded samples, that allows for the identification of the majority of albatross, petrel and shearwater species listed under ACAP and Australia's Threat Abatement Plan‐Seabirds. This method uses DNA extracted from feathers and facilitates a simple but effective way to improve data collection and quality to inform fisheries management. We also provide 275 new mitochondrial reference DNA sequences for 29 ACAP and TAP‐Seabirds listed species, which substantially improves the coverage of reference databases.

For our 36 listed species, we show that a multi‐marker approach enables the identification of unknown specimens to species or sister species level. An advantage of using multiple markers is the increased confidence in positive detections, as it provides more than one line of evidence for the presence of a certain species (Brys et al. 2023). Although this method has been developed and optimised for the identification of seabirds of specific concern to ACAP and Australian authorities, we also provide a basis for the international application of these methods outside Australian waters, and for the identification of other procellariiform species. We have demonstrated the broad utility of our selected genetic markers for identification of all procellariiforms. Although, globally, numerous species are not well represented in our custom reference databases, 25 of the 26 procellariiform genera are included in the Cytb database (*Nesofregetta* is the exception). The results of our genetic distance‐based analysis indicate that it should be possible to identify specimens from most procellariiforms to at least genus level using these two markers, and 74% of those species with reference sequences can be identified to species or sister species level based on the current Cytb_AP and CRBird_AP databases. However, given the current lack of reference data for some taxa—including many species represented by only a single sequence—a precautionary approach is needed for application beyond our listed species.

### 4.1 Benefits of a Multi‐Marker Approach for Species Identification

In this study, the combined use of the Cytb_AP and CRBird_AP markers enabled identification of most listed species. All Southern Hemisphere, albatrosses could be assigned to species level using the CRBird_AP marker, except for the closely related sister species northern and southern royal albatross. Population genomics and other approaches also have the potential to identify genetic markers from the nuclear genome that could be used for species identification in this and similar cases (Abbott and Double 2003a).

Nearly all listed petrels and shearwaters were identified as species using a combination of Cytb_AP and CRBird_AP markers, except for the great‐winged petrel and Balearic shearwater. Although these could be differentiated from other ACAP and TAP‐Seabird species with the Cytb_AP marker, they were genetically similar to the white‐headed petrel and Yelkouan shearwater, respectively. Since we lacked CRBird_AP reference data for the great‐winged petrel and Balearic shearwater, we could not evaluate the ability of the CRBird_AP marker to distinguish these two species. Should reference data for the CR marker become available in the future, this may provide greater species resolution.

The CRBird_AP marker preferentially amplified CR copy 1 or both copies for three of the North Pacific albatrosses (*Phoebastria*): Laysan, black‐footed and short‐tailed albatrosses, and provided an example of the benefits of having a complete annotated mitochondrial genome to enable evaluation of duplicated regions. The primers used here were designed to target CR copy 2 in Southern Hemisphere albatrosses. Although the CR sequences obtained were still valid for species identification, we recommend caution if relying on the CRBird_AP marker alone for identification in this genus because of this uncertainty. The Cytb_AP marker can identify short‐tailed and black‐footed albatross to species and Laysan and black‐footed albatross to sister species. For species discrimination in *Phoebastria* sp., existing Cytb (Walsh and Edwards 2005) or CR domain 2 markers, designed specifically for *Phoebastria* sp., (Eda et al. 2010) can be applied.

### 4.2 Need for Improved Reference DNA Sequence Databases

Comprehensive reference DNA sequence databases, against which sequences from unknown specimens can be compared, are essential for species identification based on genetic markers (Guo et al. 2022). However, reference databases are typically incomplete, may contain errors, and poorly reflect intraspecific variation, even for well‐characterised taxa such as vertebrates (Furlan, Davis, and Duncan 2020). For example, no CRBird_AP reference sequences were available for the five *Procellaria* species. Fortunately, Cytb_AP and COI_AP reference sequences were available for four of these species, enabling identification with these markers, although such identifications need to be interpreted with consideration as the lack of reference sequences means spectacled petrel *(P. conspicillata*) cannot be ruled out.

This study has substantially increased the availability of mitochondrial reference DNA sequences for ACAP and TAP‐Seabirds listed species. With the combination of Cytb_AP and CRBird_AP data, reference DNA sequences are now available for all but one of the listed species. We also provide a comprehensive summary of the availability of reference DNA sequences across all procellariiforms for three mitochondrial genes, highlighting the need for additional sequencing to fill taxonomic gaps and improve knowledge of genetic within‐species variation.

The importance of generating reference sequences from known provenance specimens is widely recognised in the literature (MacDonald and Sarre 2017; Päckert 2022; Roycroft et al. 2022; van den Burg and Vieites 2023). In particular, DNA sequences from vouchered museum specimens (Buckner et al. 2021) provide clear links between genetic data and taxonomy, although it can be a challenge to obtain high‐quality DNA sequences from some historical museum specimens. In the case of seabirds, confidence in the taxonomic identification of specimens may vary between samples collected at breeding sites and samples collected from birds at sea, and this should be considered during the curation of reference sequence databases. For example, GenBank accession AY158677 was excluded from our custom database as it appeared to have an incorrect taxonomy. Putatively from a black‐browed albatross, the museum sample was collected from the Northland region of New Zealand, which is not a known breeding site of this species or the closely related Campbell albatross. The presence of a lineage‐specific CR sequence (DiC GCRGCTGG, Burg et al. 2017) suggests it should now be assigned to Campbell albatross. Birds with this unique eight‐nucleotide mitochondrial sequence occur only at Campbell Island (Burg et al. 2017); hence, the provenance of individuals of this type can be assigned with high certainty.

Given the current gaps in reference data for procellariiforms, we emphasise the need to consider other data types, such as geographic sampling location, in conjunction with genetic sequence data for those taxa. For example, some genera (such as *Puffinus*) include multiple species that currently lack reference DNA sequences. This means it will be difficult to assign an unknown specimen to a *Puffinus* species based on DNA sequence data alone unless all species without reference DNA sequences can be excluded on other grounds.

### 4.3 Custom Reference Sequence Database for ACAP Listed Species

There is a strong need for a custom database for bycatch detection and identification to meet the needs of ACAP and fishery managers. This study has encouraged ACAP to support the development of a site‐specific database of samples from known provenance specimens to improve the accuracy of future studies focused on ACAP‐listed species (Tasker et al. 2023). It has also initiated the development of a validated and curated dataset of reference sequences specifically designed for taxonomic identification.

The approach of querying unknown sequences against a custom database differs from standard BLAST searches against GenBank. GenBank is not curated and is known to include sequences with erroneous or outdated taxonomic identifications (MacDonald and Sarre 2017; Li et al. 2018; van den Burg, Herrando‐Pérez, and Vieites 2020; Sangster and Luksenburg 2021; van den Burg and Vieites 2023). In the case of bird specimens, this may occur because some species can be easily misidentified; the diagnostic morphological features used to distinguish species can be subtle.

The custom reference sequence databases developed during this study will be made available for species identification of unknown DNA sequences for these genetic markers, using the Web‐based software DNA Surveillance (Ross et al. 2003; https://dna‐surveillance.auckland.ac.nz/). Using this software, unknown sequences can be aligned against a custom database of sequences from known species, and results are returned in the form of a phylogenetic tree. Despite our efforts, the three reference databases developed during this study remain incomplete. We were unable to generate sequences from samples of known provenance for some species or to obtain samples or data for others. However, the creation of this custom database provides a foundation to increase the number of samples of known provenance and to improve our ability to detect inter‐ and intraspecific variability, which is currently limited by a low number of sequences available per species. It will be important to re‐evaluate the methods outlined in this study as more reference DNA sequences become available in the future through this database and to update recommendations as needed.

### 4.4 Mitochondrial Genome Complexity in Seabirds

The complexity and variation within the mitochondrial genomes of procellariiforms can impede the development of validated markers for species identification. The Control Region is recognised as being particularly complex in procellariiforms. Abbott et al. (2005) observed a mitochondrial duplication including the CR in five albatross species. Subsequently, similar mitochondrial duplications have been observed in other procellariiform species (see Torres et al. 2019 for a summary of mitochondrial duplications in procellariiforms). These duplications complicate PCR and sequencing analyses because amplification and sequencing of markers within the duplicated region risks co‐amplification of multiple paralogues. Here, we used a copy‐specific primer, designed for southern hemisphere *Thalassarche* and *Diomedea* albatrosses (Abbott et al. 2005; Rains, Weimerskirch, and Burg 2011) to amplify CR copy 2. However, some of our CRBird_AP Sanger sequences still included a small number of base ambiguities, suggesting the primers occasionally amplified two slightly different products.

Further, we attempted to amplify and sequence our selected CRBird_AP marker from white‐chinned and grey petrels using the CRBird primers, but gel electrophoresis showed either two bands were co‐amplified (355 and 750 bp; *Procellaria aequinoctialis* ) or one large band (750 bp, *P. cinerea* ). This suggests these primers amplified two copies of the control region in *P. aequinoctialis* , and are unsuitable for sequencing unless each band is extracted from the gel. No other reference sequences exist for the CRBird_AP in *Procellaria*, so we were unable to evaluate the conservation of primer binding sites in CR copy 1 or 2. Taken together, these examples highlight the need to resolve mitochondrial structure, especially concerning the control region, in all procellariiform genera. In future work, the use of long‐read sequencing methods to develop whole mitochondrial genomes for all ACAP‐ and TAP‐Seabird species is likely to provide better resolution of mitochondrial genome duplications, enable the development of more complete reference databases and might identify additional markers for species identification.

### 4.5 The Application of Molecular Methods to Understanding Seabird Bycatch

The discrepancies between genetic and e‐log records are not surprising and highlight that the identification of seabird carcasses is difficult. This result also emphasises the need for alternative methods to obtain reliable bycatch data that do not rely on the presence of skilled observers on fishing vessels. The AAD and AFMA aim to establish an efficient and effective protocol for species identification and reporting of seabird bycatch in TAP fisheries. Implementing the protocol will help in reviewing the information provided by fishing operators, for example, by comparing species identifications from electronic monitoring footage and feather DNA for bycaught seabirds with the species identification in logbook returns received by AFMA. We acknowledge that it's not always possible to collect a feather sample from every dead seabird reported in e‐logs (e.g. the carcass might come off the line before retrieval on board). Although feather collection has been compulsory in TAP‐Seabird fisheries since 2020, at this stage genetic testing has only been applied to 56 feathers collected from the ETBF and three from the GHAT sector of the SESSF. Between 2019 and 2021, 153 dead seabirds were reported in the ETBF, and 39 feather samples (25%) were submitted for genetic analysis. Increased awareness of this issue within the fishery, and outreach by AFMA, led to an increase in feathers submitted in 2022, representing 35 (60%) of the 58 dead seabird interactions recorded in that year for the ETBF and three (15%) of the 20 in the GHAT sector of the SESSF. AFMA has assessed the potential risk of the current avian flu outbreak to their fishing operators and the feather programme is now being undertaken on a voluntary basis. This will impact future feather collections, hampering the wider implementation of these methods.

Our study highlights the prevalence of high‐risk species bycaught in Australian waters, including flesh‐footed shearwaters, Antipodean and white‐capped albatrosses. Genetic results indicate that 51 of 59 (86%) feather samples analysed from 2019 to 2022 from the ETBF fishery belonged to these three threatened species. However, these feather samples represented only 24% of the overall seabird bycatch deaths (*n* = 211) during this time. If the feather samples analysed are representative of the overall species composition of bycatch, there is a reason for concern due to the disproportionate representation of these three species. Previous bycatch data from the ETBF collected between 2001 and 2006, from 280 specimens retained for necropsy, were dominated by flesh‐footed shearwaters (78%, Trebilco et al. 2010). Smaller numbers of albatrosses made up the remainder of the bycatch (eight wandering, six black‐browed and four shy albatrosses; Trebilco et al. 2010). Data from these studies improve our understanding of the potential ongoing risks for these species in Australian waters. The threats to and impacts of anthropogenic activities such as longlining on albatrosses are especially serious given the limited capacity of albatross populations to cope with increased levels of mortality (Phillips et al. 2016; Petrossian et al. 2022). These genetic methods also allow us to determine the sex of bycaught birds, which enables improved estimates of risk and informs population models. Some albatross and petrel species are known to have sex‐specific differences in foraging strategies, which can expose one sex to increased risk from fisheries (Gianuca et al. 2017; Reyes‐González et al. 2021). In this study, there was no difference in the sex ratio of flesh‐footed shearwaters: however, there was a strong bias towards bycatch of male Antipodean albatross. These data are integral to monitoring and assessing the impacts of fisheries on population trends. The assignment of sex in bycatch assessment has been recommended (Gianuca et al. 2017), however obtaining these data can be challenging visually. The use of genetic methods would address these current challenges.

Understanding fishery impacts at the subspecies or population level will also be important. Currently, ACAP does not consider *D. a. antipodensis* and *D. a. gibsoni* as separate species, although other taxonomies do. Burg (2023) was able to distinguish these two subspecies using new analyses of nine previously genotyped microsatellite markers. In that study, bycatch from two locations (46° S 175° E in April 1997, and, 37° S 179° E in July 1997) comprised only *D. a. antipodensis*. The 18 bycatch feathers identified as Antipodean albatross in this study were all caught from early September to late October, at 26° S to 30° S, highlighting the relatively localised spatial and temporal period of bycatch of these species for ETBF. More information is needed to identify what additional bycatch mitigation is required to reduce the risk to these taxa in this region, and perhaps a greater definition of species‐specific triggers for fisheries to initiate greater management action, e.g. bycatch limits.

## 5 Conclusions

This paper provides a standardised approach to detecting seabird bycatch in Australian fisheries and a step towards a more global approach for detecting all procellariiforms bycatch species. While no single marker was able to identify all of the listed procellariiforms to species we suggest the following workflow. For an unknown sample from Australian waters, we recommend using Cytb_AP and CRBird_AP for species identification. If laboratory resources are limited we recommend first sequencing with Cytb_AP and if species resolution is insufficient, then sequence with CRBird_AP (Figure 3). The CRBird_AP markers biggest strength is species identification in albatrosses, but it is also hindered by double copies which vary within the procellariiforms. We recommend the development of a collection of genetic samples of known provenance from all ACAP and TAP‐Seabird listed species, as well as their close relatives, and that these are used to expand reference DNA sequence databases, potentially including full mitochondrial genomes. While our focus here has been on Australian fisheries, the standardised inclusion of genetic methods similar to those presented here could be included in monitoring conducted by other nations and Regional Fisheries Management Organisations (RFMO). However, further work is needed to ensure procellariiform reference sequence databases are complete and accurate. The above workflow will need to be reassessed once missing reference sequences are obtained to ensure validity for all procellariiform species. This will improve the ability to determine species‐level impacts of fishing operations on seabirds and particularly threatened albatrosses and petrels globally.

The combined use of the Cytb_AP and CRBird_AP markers provides an easily applied, simple, and effective genetic tool to identify seabird species using DNA extracted from feathers, while genetic sex identification provides additional benefit with minimal additional effort. The results from our case study highlight the difficulty and inaccuracies associated with the visual identification of bycatch species. These genetic methods have the potential to significantly augment existing bycatch monitoring methods and improve confidence in our understanding of species‐level impacts by specific fisheries, providing accurate identification of impacted species as required in the TAP. This study has also highlighted the prevalence of threatened species caught as bycatch in Australian waters and the need for improved mitigation measures to reduce seabird mortality and improve conservation outcomes for these threatened species.

## Author Contributions

**Andrea M. Polanowski:** conceptualization (equal), data curation (equal), investigation (lead), resources (equal), validation (lead), writing – original draft (lead), writing – review and editing (equal). **Anna J. MacDonald:** data curation (equal), formal analysis (lead), resources (equal), visualization (equal), writing – original draft (lead), writing – review and editing (equal). **Mike C. Double:** conceptualization (equal), resources (equal), writing – original draft (supporting), writing – review and editing (supporting). **Jonathon H. S. Barrington:** conceptualization (equal), writing – original draft (supporting), writing – review and editing (supporting). **Theresa M. Burg:** resources (equal), writing – original draft (supporting), writing – review and editing (supporting). **Barbara Wienecke:** conceptualization (equal), writing – original draft (supporting), writing – review and editing (supporting). **Julie C. McInnes:** resources (equal), visualization (lead), writing – original draft (lead), writing – review and editing (equal).

## Conflicts of Interest

The authors declare no conflicts of interest.

## Supporting information


Data S1

Tables S1‐S3

## Data Availability

Reference DNA sequences are available on GenBank (accession numbers: PP412076—PP412170 and PP447552—PP447727). Custom reference DNA sequence databases for procellariiforms for each marker in FASTA format are available as Supporting Information (SuppInfo_COI_AP_reference_database.txt, SuppInfo_Cytb_AP_reference_database.txt and SuppInfo_CRBird_AP_reference_database.txt). DNA sequences obtained from 59 feathers samples from bycaught seabirds using COI_AP (*n* = 19), Cytb_AP (*n* = 59) and CRBird_AP (*n* = 54) markers in FASTA format are available as Supporting Information (SuppInfo_Bycatch_feather_sequences.txt). The number of sequences and unique haplotypes included in the custom reference databases for all procellariiform species, and the resolution of each marker for species identification, for each of three genetic markers: COI_AP, CRBird_AP and Cytb_AP are available as Supporting Information (Table S2). All of the supporting information has also been added to the Australian Antarctic Data Centre (doi: 10.26179/073w‐f172).

## Appendix 1 Extended Methods

### Development of Custom Reference DNA Sequence Databases for Listed Species and for All Procellariiforms

Correct taxonomic assignment of the listed species depends on the existence and the quality of genetic databases (Conde‐Sousa, Pinto, and Amorim 2019). Reference DNA sequences should ideally be sourced from samples of known provenance (e.g. samples collected from breeding sites) that have reliable taxonomic identification. We assessed the availability of procellariiform mitochondrial DNA reference sequences from the NCBI GenBank database.

Initially, to inform primer design and evaluation of the six genetic markers described above, we retrieved all available COI, CR and Cytb sequences for the 36 listed species (GenBank accessed in March 2023). For each gene region, sequences were aligned in Sequencher (version. 4.10.1) and manually inspected for conserved regions.

Following marker selection, we identified gaps in reference sequence coverage for the 36 listed procellariiform species. To address these data gaps, we sourced 99 reference samples from DNA, tissue, blood or feathers from listed procellariiform species.

#### Collection of Reference Samples

Reference samples were obtained from museum collections, archived samples at the AAD, samples collected from wild populations and bycaught seabirds that could be reliably identified (e.g. by experienced seabird biologists, or the presence of leg bands). Of the 99 reference samples, 18 were from the Tasmanian Museum and Art Gallery (TMAG); 27 were collected in the wild from feathers (*n* = 5), blood (*n* = 12) and deceased seabirds (*n* = 10); 30 were tissues from seabird bycatch from fishing grounds off Tasmania (Australia, *n* = 7), Chatham Rise (New Zealand, *n* = 8), Alaska (USA, *n* = 3) and Hawaii (USA, *n* = 12); and 24 were from banded birds from either the Southern Ocean Seabird Study Association (SOSSA, *n* = 18), chick banding on Macquarie Island (*n* = 2), deceased seabirds (*n* = 2), or bycatch (*n* = 2). All samples included in this study were accessed with the permission of the relevant collection manager or custodian (see Table S1 for collection details for each sample). Destructive sampling from TMAG specimens was approved under request SR096 (June 2021). The Australian Bird and Bat Banding Scheme (ABBBS) and the New Zealand National Bird Banding Scheme (NZNBBS) were able to provide banding information and re‐sighting data for some of these individual birds. In total, the reference samples represented 29 of the 36 listed species (81%) and where possible included samples from multiple individuals and/or locations per species (Table S1).

#### DNA Extraction From Reference Samples

DNA from 84 reference samples was prepared for sequencing in the AAD's laboratories. DNA was extracted from 18 museum samples and five feather samples using the Qiagen DNeasy Blood & Tissue kit (Qiagen), with modifications based on Joseph et al. (2016). The extractions were carried out in a separate clean laboratory, which was physically separated from the main genetics laboratory. These 23 DNA extractions, plus an additional 61 DNA samples (which had been previously extracted using a range of methods), were quantified using Qubit 2.0 Fluorometer and the Qubit dsDNA broad range (BR) assay kit (Life Technologies).

#### Sanger Sequencing of Reference Samples

The 84 reference DNA samples extracted at the AAD were plated out at 5 ng/μL and sent to the Garvan Institute of Medical Research (https://www.garvan.org.au/) for PCR amplification and dual‐direction Sanger sequencing using the three primer pairs in Table 1. Each PCR mix contained 0.5 μM each of the relevant forward and reverse primers, 12.5 μL AmpliTaq Gold™ 360 Master Mix in 1× reaction buffer (Life Technologies) and 2 μL DNA extract in a total reaction volume of 25 μL. Thermal cycling conditions for Cytb_AP and CRBird_AP were 95°C for 10 min, followed by 35 cycles (40 cycles for feather and museum samples) of 95°C for 30 s, annealing temperature (53°C Cytb_AP, 50°C CRBird_AP) for 30 s and 72°C for 45 s, with a final extension of 72°C for 7 min. For COI_AP, the first round was the touchdown protocol as per Leray et al. (2013), namely 95°C for 10 min, a 16‐cycle touchdown phase (62°C–1°C per cycle), followed by 25 cycles with an annealing temperature of 46°C (total of 41 cycles) and a final extension at 72°C for 5 min. Negative controls included one extraction blank (DNA extraction without a sample) and one PCR blank (no DNA added to the PCR mix). The PCR products of all reference samples (and negative controls) were Sanger sequenced in both directions.

Reference DNA sequences for an additional 15 samples from the U.S. Fish and Wildlife Service (USFWS) were obtained through collaboration with B. N. Sacks, from the University of California, and his colleagues E. Pulido and S. Vanderzwan. The three primer pairs used in this study were sent to the University of California and their 15 DNA samples were sequenced according to the above protocol. Two CR copy 2 sequences, previously unpublished by Rains, Weimerskirch, and Burg 2011, were also included in this study (Table S1; *Diomedea exulans*; PP712121 and PP712122).

Sequences were trimmed, edited, and aligned using GeneiousPrime 2022.0.1 (https://www.geneious.com) and queried (blastn) against the nucleotide database of the National Center for Biotechnology Information (NCBI) to confirm the identification of each sequenced PCR product (Table S1).

#### Compilation of Reference DNA Sequence Databases for All Procellariiforms

To enable evaluation of the three selected markers across all procellariiform species, we subsequently developed a custom reference DNA sequence database for each marker (COI_AP, Cytb_AP and CRBird_AP), using all available sequences from all 149 procellariiform species. For each of the three genetic markers, we accessed all relevant sequences available from GenBank for all procellariiform species (families *Diomedeidae*, *Hydrobatidae*, *Oceanitidae* and *Procellariidae*; accessed July 2023). We also extracted the relevant sequences of the mitochondrial genomes assembled for four North Pacific albatross species (genus *Phoebastria*) and for wandering albatross (*D. exulans*) by Huynh et al. (2023) and included the new reference sequences generated in this study (described above).

For the Control Region marker CRBird_AP, GenBank sequences identified as originating from CR copy 1 were excluded from the reference database. The CRBird_R primer binding site is conserved in both CR copy 1 and CR copy 2 of the available reference sequences for the species considered in this study. However, the CRBird_F primer was designed to specifically amplify CR copy 2, and the binding site for this primer is not conserved in CR copy 1 sequences. This means that PCRs using the CRBird F and R primers are expected to amplify only the CR copy 2 sequence. It was not clear whether some GenBank sequences originated from CR copy 1 or 2: in these cases, a sequence was only included in the reference database if the CRBird_F primer binding site could be identified, as expected, within that sequence.

Sequences were downloaded into GeneiousPrime 2022.0.1 (https://www.geneious.com) and a custom database was constructed for each of the three markers in turn by aligning all relevant sequences (including all GenBank sequences and the Sanger sequences from listed species generated in this study), trimming the alignments to the amplicon region, and reviewing sequence quality and coverage. Sequences were aligned using the Geneious alignment method to automatically determine sequence direction. Each resulting alignment was annotated with the relevant forward and reverse primer sequences (with up to 5 mismatches allowed) to identify the amplicon region. The alignment was trimmed to exclude any parts of each sequence outside the amplicon region so that all sequences within the alignment (including any gaps) were the same length. Individual sequences were excluded if they could not be aligned to the amplicon region (e.g. GenBank sequences representing different fragments of the same gene), did not span the full length of the amplicon, or contained unidentified bases (Ns). Any uracil bases in GenBank sequences derived from RNA were converted to thymine bases to enable subsequent analyses. Additionally, the CRBird_AP alignment was trimmed to exclude the nine nucleotides immediately adjacent to the CRBird_R primer, and the COI_AP alignment was trimmed to exclude the three nucleotides immediately adjacent to the Leray_R primer, in each case to enable the inclusion of several GenBank sequences that were slightly shorter than our intended amplicon. Following trimming and quality control, each alignment was realigned using the Geneious alignment method, in case the removal of incomplete or lower quality sequences resolved any gaps. Each alignment was exported from Geneious as a fasta file (SuppInfo_COI_AP, SuppInfo_Cytb_AP, SuppInfo_CRBird_AP).

### In Silico Evaluation of Markers for Identification of Listed Species and All Procellariiform Species

Using a genetic distance‐based method we evaluated the utility of the three selected genetic markers, for species‐level identification of the 36 listed species. We also evaluated the three markers for a potentially broader application to identify all procellariiform species at risk of misidentification. We used the R package SPIDER (Brown et al. 2012), using the custom databases we had developed for all procellariiform species (described above) as the three input data files. Species with only one unique haplotype observed were included in these analyses, but intraspecific genetic distances cannot be evaluated for these species, which limits some interpretation of the results. For each marker, pairwise genetic distance was calculated for each pair of sequences using the ‘raw’ or uncorrected model (Collins et al. 2012; Srivathsan and Meier 2012). We then analysed each database using the *threshID* function to identify instances where a risk of species misidentification or ambiguity was likely, and to identify genetic distance thresholds that might be used to guide the assignment of DNA sequences of unknown provenance to a species or genus.

The *threshID* analysis tests, for each sequence in the custom database in turn, whether other sequences in the database, that are within a specified genetic distance threshold, originate from the same species or genus as the query sequence. For example, a threshold of 1% considers only sequence pairs that are within 1% genetic distance of each other, that are at least 99% identical. Here, we evaluated genetic distance thresholds for each marker from 1% to 10%, with increments of 0.5%. Four outcomes were possible for each query: ‘correct’ means that all other sequences within the threshold were conspecific; ‘incorrect’ means that all other sequences within the threshold were from a different species; ‘ambiguous’ means that other sequences within the threshold originated from more than one species, including conspecifics; and ‘no identification’ means that no other sequences in the reference database were within the specific genetic distance threshold. The same analysis was also conducted at the genus level, which is to evaluate the ability to distinguish the different genera with these genetic markers. From this, we determined the proportion of species that could be assigned to species or sister species for each marker, both for the procellariiform order overall as well as within each of the four procellariiform families.

### Case Study: Genetic Identification of Listed Species From Bycatch Feather Samples

#### Collection of Bycatch Feathers

Feather samples were collected from 59 seabirds caught incidentally from 2019 to 2022 (56 in the ETBF and 3 in the GHAT sector of the SESSF). Multiple feathers were plucked from each deceased bird following established protocols and stored at −20°C until DNA could be extracted. DNA was extracted at the Australian Genome Research Facility (AGRF; http://www.agrf.org.au) from 1 to 3 feather quill tips per sample, using the NucleoSpin® Tissue system (Macherey‐Nagel GmbH & Co. KG, 52355, Düren, Germany) as per the manufacturer's protocol for tissue samples. Purified DNA was quantified via UV absorbance (NanoDrop ND‐8000 Spectrophotometer; Thermo Fisher Scientific, Waltham, MA USA) and diluted to 5 ng/μL.

#### Sanger Sequencing of Bycatch Feathers

The bycatch feathers were processed in two groups. Group one was a trial group and included 20 feathers collected from 2019 to 2021. These samples were amplified with all three markers (COI_AP, CRBird_AP, and Cytb_AP). Group two included 39 feathers collected in 2022. These samples were amplified at the AAD genetics laboratories with the two markers recommended based on the results of the initial trials: CRBird_AP and Cytb_AP. PCR products were purified using Agencourt AMPure XP beads (Beckman Coulter) and quantified with Qubit 2.0 Fluorometer using the Qubit dsDNA high‐sensitivity (HS) assay kit (Life Technologies). For the sequencing reaction, PCR products were diluted in water according to fragment size (6–12 ng DNA, 200–400 bp product) and 10 pmol of forward or reverse primer was added in a total of 12 μL reaction volume. The samples (including negative controls) were then sent to AGRF for Sanger sequencing in both directions. Sequences were trimmed, edited and aligned using GeneiousPrime 2022.0.1 (https://www.geneious.com) and queried against NCBI and custom sequence databases to confirm the identification of the sequenced PCR product.

AFMA e‐log records were available for the 59 feathers, which include seabird identifications based on visual observation by the fishery operators.

#### PCR Sexing of Bycatch Feathers

For each of the bycatch specimens (*n* = 59), sex was also determined by analysis of feather DNA using a real‐time melt curve analysis (Faux, McInnes, and Jarman 2014). The PCR mix contained 1 μM for each forward (PenF1‐CAGCTTTAATGGAAGTGAAGG) and reverse primer (PenR2‐GGAGTCACTATCAGAYCC), 1× LightCycler 480 Probes Master (Roche), 1× EvaGreen (Biotium) and 2 μL DNA extract, in a total reaction volume of 10 μL. Thermal cycling conditions were 95°C for 5 min; followed by 40 cycles of 95°C for 10 s, 52°C for 30 s and 72°C for 10 s. Melt curve conditions were 55–95°C at a ramp rate of 2.2°C/s with 5 acquisitions per degree. PCR controls included DNA from six known females (*Ardenna tenuirostris*, *Diomedea antipodensis*, *T. bulle*r*i*, *T. cauta*, *T. melanophris* and *T. steadi*), four known males (*A. tenuirostris*, *A. creatopus*, *D. antipodensis* and *T. steadi*) and a negative.

## Appendix 2 Extended Results

### In Silico Evaluation of Markers for Identification of Listed Species and All Procellariiform Species

In the *threshID* analyses at the genus level, a cumulative error was lowest at a 4% threshold for the COI and Cytb markers, and a 7% threshold for the CR marker. In other words, if a sequence from an unknown sample has > 96% identity (COI and Cytb) or > 93% identity (CR) with known sequences from a single genus in the relevant reference database, this should provide sufficient confidence in the identification at the genus level. The errors observed at these thresholds were all in the ‘no identification’ category, that is the query sequences had < 96% (COI and Cytb) or < 93% (CR) identity with any other sequences in the reference database (Table A5). This means it is more likely that an unknown sequence would not be assigned to any genus, rather than mis‐assigned to an incorrect genus. In many cases, the sequences that could not be identified were the only representative sequences available for the species or genus, and so were expected to be divergent from all other sequences. Increasing the availability of reference sequences to include multiple representatives of each species would reduce the risk of this class of error.

In the species‐level analyses, which evaluated all available procellariiform reference sequences for each marker, thresholds of 1% to 2% had the lowest cumulative errors. Given the lengths of the markers, a threshold of 1% could equate to a difference of only 2 or 3 nucleotides across the amplicon length. This introduces a risk of misidentification because of factors such as sequencing error when using a 1% threshold. For this reason, we focus on evaluating the risks of misidentification using a 1.5% threshold for all three markers. While many procellariiform sequences were correctly assigned to species using a 1.5% threshold, ambiguous, incorrect and ‘no ID’ results were observed for all three markers (Table A5).

These results demonstrate that the markers have different utility for different families. For COI_AP and Cytb_AP markers, risks of misidentification relevant to our listed species included ambiguities *within Ardenna, Diomedea, Macronectes, Phoebastria* and *Thalassarche* genera. In contrast, the risks of misidentification of listed species using the CRBird_AP marker were limited to ambiguities between two pairs of sister species (*Diomedea epomophora* / *sanfordi* and *Thalassarche cauta/steadi*), although this marker could not be evaluated for all petrels or shearwaters.

**TABLE A1:** Summary of the six primer pairs experimentally tested to determine amplification success in the 36 listed albatross and petrel species.

Locus	Primer Name	Primer Sequence	PCR Temp	Length (bp)	Reference	Used in current study
COI	AvMiF1 jgHCO2198R	CNCCYGAYATRGCATTYCCACG TAIACYTCIGGRTGICCRAARAAYCA	51°C	466	Modified from Kerr et al. (2009) Geller et al. (2013)	No—PCR from 7/20 feathers failed, suggesting that the amplicon length was too long for degraded samples
COI	BirdCOIF jgHCO2198R	GGNACMGGRTGRACHGTNTAYCCNCC TAIACYTCIGGRTGICCRAARAAYCA	57°C	367	Modified from Rubbmark et al. (2018) Geller et al. (2013)	Yes – was used in current study
Cytb	Cytb1‐F Cytb1‐R	GCHTGATGRAACTTYGGVTC CCTCARAAYGATATYTGBCCTC	55°C	313	This study	No—Reverse Sanger sequence failed in 7/12 feathers
Cytb	538F 956R	TTYGCYCTACAYTTYCTCCT TGRGARAGBGGRCGRAA	50°C	431	This study	No—Co‐amplified the Cytb duplication in albatross. Forward Sanger sequence had mixed traces.
Cytb	Cytb2‐F Cytb2‐R	TAYATYGGCCARACCYTYGTAG GTTYTCTGGRTCDCCKARYA	53°C	305	McInnes et al. (2021)	Yes—was used in current study
CR_ Copy 2 Domain1	CRBird_F CRBird_R	CAGCCTATGTGTTGATGTGCA CGGGTTGCTGATTTCTCGTG	50°C	379	This study Modified from Abbott and Double (2003b)	Yes—was used in current study

*Note:* Underlined bases are modifications made in this study to the original primer sequence.

**TABLE A2:** Forward PCR primer designed in the current study aligned with homologous sequences of control region copy one (F1) and two (F2) in 10 procellariiform species.

Name	Accession/Reference	Sequence
CRBird_F Primer	This study	‐‐CAGCCTATGTGTTGATGTGCA
SpecF1 Primer	Abbott and Double ( 2003b )	‐‐..T.....A.AA…C.
SpecF2 Primer	Abbott and Double ( 2003b )	AA..................
*Thalassarche melanophris* F1	AY158677.2	‐............A.AA…C...
*Thalassarche melanophris* F2	AY158677.2	
*Thalassarche chlororhynchos* F1	MN356342.1	.G....T.....N.NA…N...
*Thalassarche chlororhynchos* F2	MN356342.1	.G....T.....N.NA…N...
*Diomedea amsterdamensis* /*D.exulans* F1	Rains, Weimerskirch, and Burg ( 2011 )	‐T....T.....A.AA…C...
*Diomedea amsterdamensis* /*D.exulans* F2	Rains, Weimerskirch, and Burg ( 2011 )	
*Ardenna pacifica* only 1 copy present	NC_057528.1	GG..........A.A.....A..
*Ardenna carneipes* only 1 copy present	NC_057527.1	GG.....C....ACAA.......
*Macronectes giganteus* only 1 copy present	NC_085213.1	…T..TC....ACGTG…A..
*Fulmarus glacialis* only 1 copy ^a^	MN356131.1	......TC....ACG.GCA....
*Phoebastria albatrus* F1	KJ735512.1/AB276044‐46	…G........A.AA.......
*Phoebastria albatrus* F1	AB276047.1	…G........A..A.......
*Phoebastria albatrus* F2	KJ735512.1/AB276046.1	…G........A.GAG......
*Phoebastria albatrus* F2	AB276045.1	…G........A.A.G......
*Phoebastria albatrus* F2	AB276047.1	…G........A.A........
*Phoebastria albatrus* F2	AB276044.1	…G........ACGAG......
*Phoebastria nigripes* F1	KJ735512.1/AB276051/56/58/61/62	…G........A.AA.......
*Phoebastria nigripes* F2	KJ735512.1/AB276051/57/59/61/63	…G........A.GAG......
*Phoebastria immutabilis* F1	KJ735513.1	....A.......A.AA.......
*Phoebastria immutabilis* F1	AB276049/50/54	............A.AA.......
*Phoebastria immutabilis* F1	AB276048.1	............A.AA…C...
*Phoebastria immutabilis* F2	KJ735513.1/AB276048‐50,54–55	....A.T.....A.GAG......

*Note:* The variability and duplication in the CR region prevented the development of a set of ‘universal procellariiforms’ CR primers. Similarities to CRBird F are denoted by ‘.’, gaps denoted by ‘‐’.

^a^ Burg et al. 2014 have shown evidence of CR duplication in *F. glacialis*, but it was not possible to determine which of the two copies in fulmars corresponded to the F1 and F2 copies in albatross.

**TABLE A3:** Primers screened in six white‐capped (*Thalassarche steadi*) and six shy (*Thalassarche cauta)* samples to try to find fixed genetic differences between these two closely related albatross species. The white‐capped samples were collected from Disappointment Island in 2001 (see Abbott and Double 2003a, 2003b) and the shy samples were collected from Albatross Island in 2015.

Locus	Primer name	Primer sequence	PCR Temp	Length (bp)	Reference	Results
16S	16s_1_F 16s_1R	TTGCCTGTGAAACGAATCTG GCTATGTTTTTGGTAAACAGTCG	60°C	481	This study	No differences
16S	16s_Aves_F 16s_Aves_R	AAGGTAGCGCAATCAATTGTC TGTCCTGATCCAACATCGAGG	60°C	430	This study	No differences
Z43B	Z43B_F Z43B_R	CTTGAGACTAATTCCACTCC TTTACATGGCAGCYTGA	50°C	260‐ 282	Dawson, dos Remedios, and Horsburgh (2016)	Based on the principle from Lee et al. (2010) who used two sex markers to simultaneously identify avian species and sex. Both of the albatross species produced identical size fragments (234/228 bp)
CHD	P2 P8	TCTGCATCGCTAAATCCTTT CTCCCAAGGATGAGRAAYTG	50°C	431	Griffiths et al. (1998)	As above. Both of the albatross species produced identical size fragments (332/334 bp)
Nuclear Loci: Pema 2, 3, 4, 5, 6, 7, 9, 10, 11, 12, 13 and 14	Multiple	Multiple	55°C	625 bp average	Silva, Duarte, and Coelho (2011)	Pema 5, 6, 12, 13 and 14: no fixed differences. Pema 2,3,4,7,9,10,11 failed.
Nuclear Loci: Occa 1, 2, 3, 5, 6, 7, 8, 9,10, 11 and 12	Multiple	Multiple	55°C	657 bp average	Silva, Silva, and Manuela Coelho (2012)	To compare to Abeyrama et al. (2021) Occa9 Shy—83% GG, 17% GA, 0% AA Occa9 White‐capped – 17% GG, 50% GA,33% AA Occa11 Shy—83%AA, 17% CA, 0% CC Occa11 White‐capped—0%AA, 33% CA, 67% CC Occa 3, 5, 6, 8 and 12 failed

**TABLE A4:** The number of procellariiform sequences included in the final three reference databases, one for each genetic marker, including the number of sequences per species and the number of unique haplotypes per species.

	COI_AP	Cytb_AP	CRBird_AP
*N* sequences in database	1005	2282	153
*N* species in database	93	133	38
*N* genera in database	26	25	14
Mean number (and range) of sequences per species	11 (1–152)	17 (1–192)	4 (1–35)
Mean number (and range) of haplotypes per species	2.7 (1–33)	3.1 (1–16)	3.2 (1–15)
Alignment length including gaps (bp)	310	261	361
Range of individual sequence lengths (bp)	308–310	261	304–334

**TABLE A5:** Results of the *threshID* analyses for each marker at the genus level (using a 4% genetic distance threshold for markers COI_AP and Cytb_AP, and a 7% genetic distance threshold for the CRBird_AP marker) and at the species level (using a 1.5% genetic distance threshold for all markers). For each marker, the number of sequences with correct, ambiguous, incorrect and ‘no ID’ results are shown.

Marker	Taxonomic level	Correct result	Ambiguous result	Incorrect result	‘No ID’ result
COI_AP	Genus	994	0	0	11
	Species	685	295	5	20
Cytb_AP	Genus	2274	0	0	8
	Species	985	1225	22	33
CRBird_AP	Genus	146	0	0	7
	Species	112	6	0	35

**TABLE A6:** Species resolution for the three tested primer sets for all 149 procellariiforms.

Species	Marker			Species	Marker		
	Cytb	CR	COI		Cytb	CR	COI
*Diomedea antipodensis*	Genus	Sp	Genus	*Fulmarus glacialis*	Sp	Sp	Sp
*Diomedea dabbenena*	Genus	Sp	Genus	*Fulmarus glacialoides*	Sp	X	Sp
*Diomedea epomophora*	SS	SS	SS	*Halobaena caerulea*	Sp	X	Sp
*Diomedea exulans*	Genus	Sp	Genus	*Macronectes giganteus*	SS	Sp	SS
*Diomedea sanfordi*	SS	SS	SS	*Macronectes halli*	SS	Sp	SS
*Phoebastria albatrus*	Sp	Sp	Sp	*Pachyptila belcheri*	Genus	X	Genus
*Phoebastria immutabilis*	SS	Sp	SS	*Pachyptila crassirostris*	SS	X	SS
*Phoebastria irrorata*	Sp	X	Sp	*Pachyptila desolata*	Genus	X	Genus
*Phoebastria nigripes*	SS	Sp	SS	*Pachyptila macgillivrayi*	Genus	X	X
*Phoebetria fusca*	Sp	Sp	Sp	*Pachyptila salvini*	Genus	X	Genus
*Phoebetria palpebrata*	Sp	Sp	Sp	*Pachyptila turtur*	SS	X	SS
*Thalassarche bulleri*	Sp	Sp	Genus	*Pachyptila vittata*	Genus	X	Genus
*Thalassarche carteri*	SS	Sp	SS	*Pagodroma nivea*	Sp	Sp	Sp
*Thalassarche cauta*	Genus	Sp^	Genus	*Pelecanoides garnotii*	Sp	X	X
*Thalassarche chlororhynchos*	SS	Sp	SS	*Pelecanoides georgicus*	SS	X	SS
*Thalassarche chrysostoma*	Sp	Sp	Genus	*Pelecanoides magellani*	Sp	X	Sp
*Thalassarche eremita*	Genus	Sp	Genus	*Pelecanoides urinatrix*	SS	Genus	SS
*Thalassarche impavida*	SS	Sp	Genus	*Procellaria aequinoctialis*	Sp	X	Sp
*Thalassarche melanophris*	SS	Sp	Genus	*Procellaria cinerea*	Sp	X	Sp
*Thalassarche salvini*	Genus	Sp	Genus	*Procellaria conspicillata*	X	X	X
*Thalassarche steadi*	Genus	Sp^	Genus	*Procellaria parkinsoni*	Sp	X	Sp
*Aphodroma brevirostris*	Sp	Sp	Sp	*Procellaria westlandica*	Sp	X	Sp
*Ardenna bulleri*	Sp	X	Sp	*Pseudobulweria aterrima*	Genus	X	Sp
*Ardenna carneipes*	SS	Sp	SS	*Pseudobulweria becki*	X	X	Sp
*Ardenna creatopus*	SS	Sp	SS	*Pseudobulweria macgillivrayi*	X	X	X
*Ardenna gravis*	Sp	X	Sp	*Pseudobulweria rostrata*	X	X	Sp
*Ardenna grisea*	Sp	Sp	Sp	*Pterodroma alba*	Genus	X	SS
*Ardenna pacifica*	Sp	Sp	Sp	*Pterodroma arminjoniana*	Genus	X	X
*Ardenna tenuirostris*	Sp	Sp	Sp	*Pterodroma atrata*	Genus	X	X
*Bulweria bifax*	X	X	X	*Pterodroma axillaris*	Sp	X	Sp
*Bulweria bulwerii*	Genus	X	Genus	*Pterodroma baraui*	X	X	X
*Bulweria fallax*	X	X	X	*Pterodroma brevipes*	Sp	X	X
*Calonectris borealis*	Genus	X	Genus	*Pterodroma cahow*	Sp	X	Sp
*Calonectris diomedea*	Genus	X	Genus	*Pterodroma caribbaea*	X	X	X
*Calonectris edwardsii*	Genus	X	X	*Pterodroma cervicalis*	X	X	Sp
*Calonectris leucomelas*	Sp	X	Genus	*Pterodroma cookii*	Sp	X	Sp
*Daption capense*	Sp	Sp	Sp	*Pterodroma defilippiana*	X	X	X
*Pterodroma deserta*	SS	X	X	*Puffinus myrtae*	Genus	X	X
*Pterodroma externa*	Sp	X	Sp	*Puffinus nativitatis*	Sp	X	X
*Pterodroma feae*	SS	X	X	*Puffinus newelli*	Genus	X	X
*Pterodroma gouldi*	Sp	Genus	SS	*Puffinus opisthomelas*	Genus	X	X
*Pterodroma hasitata*	Sp	X	Sp	*Puffinus persicus*	Genus	X	X
*Pterodroma heraldica*	Genus	X	X	*Puffinus puffinus* *Puffinus puffinus*	Genus	X	Sp
*Pterodroma hypoleuca*	Sp	X	X	*Puffinus subalaris*	Sp	X	X
*Pterodroma incerta*	Sp	X	X	*Puffinus yelkouan*	SS	X	X
*Pterodroma inexpectata*	Sp	X	Sp	*Thalassoica antarctica*	Sp	X	Sp
*Pterodroma lesonni*	SS	X	SS	*Hydrobates castro*	Genus	X	Sp
*Pterodroma leucoptera*	Sp	X	X	*Hydrobates cheimomnestes*	Genus	X	X
*Pterodroma longirostris*	Sp	X	Sp	*Hydrobates furcata*	Sp	X	X
*Pterodroma macroptera*	SS	X	X	*Hydrobates homochroa*	Sp	X	X
*Pterodroma madeira*	SS	X	X	*Hydrobates hornbyi*	Sp	X	X
*Pterodroma magentae*	Sp	X	Sp	*Hydrobates jabejabe*	Genus	X	X
*Pterodroma mollis*	Sp	X	Sp	*Hydrobates leucorhoa*	Genus	X	X
*Pterodroma neglecta*	Genus	X	SS	*Hydrobates macrodactyla*	X	X	X
*Pterodroma nigripennis*	Sp	X	Sp	*Hydrobates markhami*	SS	X	X
*Pterodroma occulta*	X	X	X	*Hydrobates matsudairae*	Sp	Genus	Sp
*Pterodroma phaeopygia*	Sp	X	X	*Hydrobates melania*	SS	X	Sp
*Pterodroma pycrofti*	Sp	X	X	*Hydrobates microsoma*	Sp	X	Sp
*Pterodroma sandwichensis*	Sp	X	X	*Hydrobates monorhis*	Sp	Genus	Sp
*Pterodroma solandri*	Sp	X	X	*Hydrobates monteiroi*	Genus	X	X
*Pterodroma ultima*	Genus	X	Sp	*Hydrobates pelagicus*	Sp	X	Sp
*Puffinus assimilis*	Genus	X	Sp	*Hydrobates socorroensis*	Genus	X	X
*Puffinus auricularis*	Genus	X	Sp	*Hydrobates tethys*	Sp	X	Sp
*Puffinus bailloni*	Genus	X	X	*Hydrobates tristrami*	Sp	Genus	Sp
*Puffinus bannermani*	Genus	X	X	*Fregetta grallaria*	Sp	X	Sp
*Puffinus baroli*	Genus	X	X	*Fregetta lineata*	X	X	X
*Puffinus boydi*	Genus	X	X	*Fregetta maoriana*	Sp	X	X
*Puffinus bryani*	Sp	X	X	*Fregetta tropica*	Sp	X	Sp
*Puffinus elegans*	Sp	X	X	*Garrodia nereis*	Sp	X	Sp
*Puffinus gavia*	Sp	X	Sp	*Nesofregetta fuliginosa*	X	X	Sp
*Puffinus heinrothi*	X	X	X	*Oceanites gracilis*	Sp	X	X
*Puffinus huttoni*	Sp	X	Sp	*Oceanites oceanicus*	Sp	X	Sp
*Puffinus lherminieri*	Genus	Genus	Sp	*Oceanites pincoyae*	X	X	X
*Puffinus mauretanicus*	SS	X	X	*Pelagodroma marina*	Sp	X	Sp

*Note:* Dark green shading indicates unknown sequences can be identified to species, light green indicates unknown sequences can be identified to sister species, orange indicates unknown sequences can be identified to multiple species, and X indicates a missing reference sequence. ^Species identification was based on a single nucleotide polymorphism (SNP) in the mitochondrial control region (Abbott and Double 2003b). This method has a ~3% error in assigning species (Abbott et al. 2006).

**TABLE A7:** Summary of the bycatch feather samples collected in the Eastern Tuna and Billfish Fishery (ETBF; *n* = 56) and the Gillnet Hook and Trap Sector (GHAT) of the Southern and Eastern Scalefish and Shark Fishery (SESSF; *n* = 3) between 2019 and 2022.

Date	elog record (AFMA)	Genetics ID	Sex	Species resolution			Genetic ID/elog record comparison
				COI_AP	Cytb_AP	CRBird_AP	
03/2020	*Thalassarche melanophris*	*Thalassarche steadi*	F	*Thalassarche* multi species	*Thalassarche steadi/cauta/salvins/eremita*	*Thalassarche steadi*	P
10/2019	*Ardenna tenuirostris*	*Ardenna carneipes*	U	Failed	*Ardenna carneipes/creatopus*	Failed	P
10/2019	*Diomedeidae—undifferentiated*	*Diomedea antipodensis gibsoni*	M	*Diomedea* multi species	*Diomedea* multi species	*Diomedea antipodensis gibsoni*	P
02/2019	*Ardenna spp.—undifferentiated*	*Ardenna carneipes*	F	*Ardenna carneipes/creatopus*	Ardenna carneipes/creatopus	*Ardenna carneipes*	P
05/2019	*Diomedea exulans*	*Thalassarche steadi*	M	Failed	*Thalassarche steadi/cauta/salvins/eremita*	*Thalassarche steadi*	N
04/2019	*Diomedeidae—*undifferentiated	*Thalassarche steadi*	M	*Thalassarche* multi species	Thalassarche steadi/cauta/salvins/eremita	*Thalassarche steadi*	P
11/2019	*Diomedeidae—*undifferentiated	*Thalassarche steadi*	F	*Thalassarche* multi species	Thalassarche steadi/cauta/salvins/eremita	*Thalassarche steadi*	P
09/2019	*Diomedeidae—*undifferentiated	*Thalassarche bulleri*	M	*Thalassarche* multi species	*Thalassarche bulleri*	*Thalassarche bulleri*	P
10/2019	*Diomedeidae—*undifferentiated	*Diomedea antipodensis gibsoni*	M	*Diomedea* multi species	*Diomedea multi species*	*Diomedea antipodensis gibsoni*	P
10/2019	*Diomedeidae—*undifferentiated	*Diomedea antipodensis gibsoni*	M	*Diomedea* multi species	*Diomedea multi species*	*Diomedea antipodensis gibsoni*	P
10/2019	*Diomedeidae—*undifferentiated	*Diomedea antipodensis gibsoni*	M	*Diomedea* multi species	*Diomedea multi species*	*Diomedea antipodensis gibsoni*	P
10/2019	*Diomedeidae—*undifferentiated	*Diomedea antipodensis gibsoni*	F	*Diomedea* multi species	*Diomedea multi species*	*Diomedea antipodensis gibsoni*	P
06/2020	*Thalassarche cauta*	*Thalassarche steadi*	F	*Thalassarche* multi species	*Thalassarche steadi/cauta/salvins/eremita*	*Thalassarche steadi*	P
10/2020	*Tern*	*Sterna* sp.	U	Failed	*Sterna* sp.	Failed	P
06/2021	*Diomedeidae—*undifferentiated	*Thalassarche impavida*	F	*Thalassarche* multi species	*Thalassarche melanophris/impavida*	*Thalassarche impavida*	P
10/2021	*Diomedeidae—*undifferentiated	*Thalassarche bulleri*	F	Failed	*Thalassarche bulleri*	*Thalassarche bulleri*	P
09/2021	*Diomedeidae—*undifferentiated	*Ardenna carneipes*	M	*Ardenna carneipes/creatopus*	*Ardenna carneipes/creatopus*	*Ardenna carneipes*	P
09/2021	no AFMA record	*Ardenna carneipes*	F	*Ardenna carneipes/creatopus*	*Ardenna carneipes/creatopus*	*Ardenna carneipes*	NA
09/2021	no AFMA record	*Ardenna carneipes*	F	*Ardenna carneipes/creatopus*	*Ardenna carneipes/creatopus*	*Ardenna carneipes*	NA
09/2021	no AFMA record	*Ardenna carneipes*	F	*Ardenna carneipes/creatopus*	*Ardenna carneipes/creatopus*	*Ardenna carneipes*	NA
04/2022	*Thalassarche cauta*	*Thalassarche steadi*	F	*Thalassarche multi species*	*Thalassarche steadi/cauta/salvins/eremita*	*Thalassarche steadi*	P
10/2022	*Diomedeidae—*undifferentiated	*Diomedea exulans*	M	Never sequenced	*Diomedea* multi species	*Diomedea exulans*	P
10/2022	*Diomedeidae—*undifferentiated	*Diomedea antipodensis gibsoni*	M	Never sequenced	*Diomedea* multi species	*Diomedea antipodensis gibsoni*	P
10/2022	*Diomedeidae—*undifferentiated	*Diomedea antipodensis gibsoni*	M	Never sequenced	*Diomedea* multi species	*Diomedea antipodensis gibsoni*	P
09/2022	*Ardenna spp.—*undifferentiated	*Ardenna carneipes*	F	Never sequenced	*Ardenna carneipes/creatopus*	*Ardenna carneipes*	P
10/2022	*Diomedeidae—*undifferentiated	*Diomedea antipodensis gibsoni*	M	Never sequenced	*Diomedea* multi species	*Diomedea antipodensis gibsoni*	P
10/2022	*Ardenna spp.—*undifferentiated	*Ardenna carneipes*	M	Never sequenced	*Ardenna carneipes/creatopus*	*Ardenna carneipes*	P
10/2022	*Diomedeidae—undifferentiated*	*Diomedea antipodensis gibsoni*	M	Never sequenced	*Diomedea* multi species	*Diomedea antipodensis gibsoni*	P
10/2022	*Ardenna carneipes*	*Ardenna carneipes*	M	Never sequenced	*Ardenna carneipes/creatopus*	*Ardenna carneipes*	Y
10/2022	*Diomedeidae—undifferentiated*	*Diomedea antipodensis gibsoni*	M	Never sequenced	*Diomedea* multi species	*Diomedea antipodensis gibsoni*	P
10/2022	*Procellariidae—undifferentiated*	*Ardenna carneipes*	M	Never sequenced	*Ardenna carneipes/creatopus*	*Ardenna carneipes*	P
09/2022	*Diomedea exulans*	*Diomedea antipodensis gibsoni*	F	Never sequenced	*Diomedea* multi species	*Diomedea antipodensis gibsoni*	P
10/2022	*Procellariidae—undifferentiated*	*Ardenna carneipes*	F	Never sequenced	*Ardenna carneipes/creatopus*	*Ardenna carneipes*	P
10/2022	*Diomedeidae—*undifferentiated	*Diomedea antipodensis gibsoni*	F	Never sequenced	*Diomedea* multi species	*Diomedea antipodensis gibsoni*	P
09/2022	*Ardenna spp.—*undifferentiated	*Ardenna carneipes*	F	Never sequenced	*Ardenna carneipes/creatopus*	*Ardenna carneipes*	P
10/2022	*Ardenna spp.—*undifferentiated	*Ardenna carneipes*	F	Never sequenced	*Ardenna carneipes/creatopus*	*Ardenna carneipes*	P
10/2022	*Diomedeidae—*undifferentiated	*Diomedea antipodensis gibsoni*	U	Never sequenced	*Diomedea* multi species	*Diomedea antipodensis gibsoni*	P
09/2022	*Ardenna spp.—*undifferentiated	*Ardenna carneipes*	M	Never sequenced	*Ardenna carneipes/creatopus*	*Ardenna carneipes*	P
10/2022	*Ardenna spp.—*undifferentiated	*Ardenna carneipes*	U	Never sequenced	*Ardenna carneipes/creatopus*	*Ardenna carneipes*	P
09/2022	*Ardenna spp.—*undifferentiated	*Ardenna carneipes*	F	Never sequenced	*Ardenna carneipes/creatopus*	*Ardenna carneipes*	P
10/2022	*Ardenna spp.—*undifferentiated	*Ardenna carneipes*	M	Never sequenced	*Ardenna carneipes/creatopus*	*Ardenna carneipes*	P
10/2022	*Ardenna spp.—*undifferentiated	*Ardenna carneipes*	M	Never sequenced	*Ardenna carneipes/creatopus*	*Ardenna carneipes*	P
10/2022	*Ardenna spp.—*undifferentiated	*Ardenna carneipes*	M	Never sequenced	*Ardenna carneipes/creatopus*	*Ardenna carneipes*	P
10/2022	*Ardenna spp.—*undifferentiated	*Ardenna carneipes*	M	Never sequenced	*Ardenna carneipes/creatopus*	*Ardenna carneipes*	P
10/2022	*Ardenna spp.—*undifferentiated	*Ardenna carneipes*	F	Never sequenced	*Ardenna carneipes/creatopus*	*Ardenna carneipes*	P
10/2022	*Ardenna spp.—*undifferentiated	*Ardenna carneipes*	U	Never sequenced	*Ardenna carneipes/creatopus*	*Ardenna carneipes*	P
10/2022	*Diomedeidae—*undifferentiated	*Diomedea antipodensis gibsoni*	M	Never sequenced	*Diomedea* multi species	*Diomedea antipodensis gibsoni*	P
09/2022	*Diomedeidae—*undifferentiated	*Diomedea antipodensis gibsoni*	M	Never sequenced	*Diomedea* multi species	*Diomedea antipodensis gibsoni*	P
10/2022	*Ardenna spp.—*undifferentiated	*Ardenna carneipes*	U	Never sequenced	*Ardenna carneipes/creatopus*	*Ardenna carneipes*	P
10/2022	*Diomedeidae—*undifferentiated	*Diomedea antipodensis gibsoni*	F	Never sequenced	*Diomedea* multi species	*Diomedea antipodensis gibsoni*	P
10/2022	*Ardenna spp.—*undifferentiated	*Ardenna carneipes*	M	Never sequenced	*Ardenna carneipes/creatopus*	*Ardenna carneipes*	P
09/2022	*Ardenna carneipes*	*Ardenna carneipes*	M	Never sequenced	*Ardenna carneipes/creatopus*	*Ardenna carneipes*	Y
10/2022	*Ardenna spp.—*undifferentiated	*Ardenna carneipes*	F	Never sequenced	*Ardenna carneipes/creatopus*	*Ardenna carneipes*	P
10/2022	*Diomedeidae—*undifferentiated	*Diomedea antipodensis gibsoni*	F	Never sequenced	*Diomedea* multi species	*Diomedea antipodensis gibsoni*	P
10/2022	*Diomedeidae—*undifferentiated	*Diomedea antipodensis gibsoni*	M	Never sequenced	*Diomedea* multi species	*Diomedea antipodensis gibsoni*	P
12/2022	*Ardenna tenuirostris*	*Procellaria aequinoctialis*	F	Failed	*Procellaria aequinoctialis*	Failed	N
12/2022	*Ardenna tenuirostris*	*Procellaria aequinoctialis*	M	Failed	*Procellaria aequinoctialis*	Failed	N
12/2022	*Procellariidae—undifferentiated*	*Ardenna carneipes*	F	Ardenna carneipes/creatopus	*Ardenna carneipes/creatopus*	*Ardenna carneipes*	P
12/2022	*Ardenna tenuirostris*	*Procellaria aequinoctialis*	F	*Procellaria aequinoctialis*	*Procellaria aequinoctialis*	Failed	N

*Note:* Visual observation data provided by AFMA, extracted from e‐logbooks. Sequences were generated using the following primers – COI_AP (BirdCOIF/jgHCO2198R); Cytb_AP (Cytb2‐F/Cytb2‐R); CRBird_AP (CRBird_F/CRBird_R). Species resolution was assessed for each marker based on results from Figure 2. Some feathers from 2022 were not sequenced with COI_AP markers. Sex: M is Male, F is Female and U is unknown. Logbook ID/elog record agreement: N for not concordant, Y for concordant and P for partially concordant.
